# Integration of 2D Materials in Radial van der Waals Heterostructure Metasurfaces

**DOI:** 10.1021/acsnano.5c20740

**Published:** 2026-05-21

**Authors:** Connor Heimig, Jonas Biechteler, Cristina Cruciano, Armando Genco, Thomas Weber, Michael Hirler, Dmytro Gryb, Leonardo de S. Menezes, Gianluca Valentini, Cristian Manzoni, Giulio Cerullo, Stefan A. Maier, Alexander A. Antonov, Luca Sortino, Andreas Tittl

**Affiliations:** † Chair in Hybrid Nanosystems, Nanoinstitute Munich, Faculty of Physics, 9183 Ludwig-Maximilians-University, 80539 Munich, Germany; ‡ Dipartimento di Fisica, 274268 Politecnico di Milano, 20133 Milano, Italy; § Dipartimento di Fisica, Università di Pisa, 56127 Pisa, Italy; ∥ Departamento de Física, 28116 Universidade Federal de Pernambuco, 50670-901 Recife-PE, Brazil; ⊥ IFN-CNR, 96976 Istituto di Fotonica e Nanotecnologie, 20133 Milano, Italy; # School of Physics and Astronomy, 2541 Monash University, Melbourne, VIC 3800, Australia; + Department of Physics, Imperial College London, London, SW7 2AZ, United Kingdom; & Institute of Photonics, Hamburg University of Technology, 21073 Hamburg, Germany

**Keywords:** van der Waals heterostructures, metasurfaces, bound states in the continuum, low-index photonics, exciton-photon coupling

## Abstract

Two-dimensional semiconductors, such as monolayer transition metal dichalcogenides (TMD), exhibit strong excitonic transitions at room temperature and offer a platform for exploring light-matter interactions in nanoscale photonic systems. In this work, we demonstrate a compact and polarization-invariant photonic metasurface, fabricated from hexagonal boron-nitride (hBN) and based on radial bound states in the continuum (BIC), which are formed by radially distributed pairs of structurally asymmetric resonators. The metasurface employs multiple symmetry-breaking perturbations to support high-quality (*Q*) factor resonances within a radial footprint of 4.5 μm – approximately one-sixth of the area of previous hBN BIC metasurface implementations based on large periodic arrays. Compared to these approaches, the radial geometry furthermore achieves sizable *Q*-factors with a reduced footprint. By integrating the hBN photonic structure with a WS_2_ monolayer, we observe enhanced photoluminescence when its resonance is spectrally aligned with the exciton resonance, accompanied by signatures of discrete momentum-space patterns that identify the orbital-angular-momentum-carrying ring eigenmodes. These features persist over a wide range of excitation powers and show minimal linewidth broadening, indicating robust and spatially modulated exciton-photon coupling. This work establishes a scalable approach for generating hybrid photonic-excitonic states with momentum-space structure, offering opportunities for exciton localization, valley emission, spatially programmable light-matter interaction in 2D material platforms and compact luminescent devices based on 2D material integrated metasurfaces.

## Graphical abstract

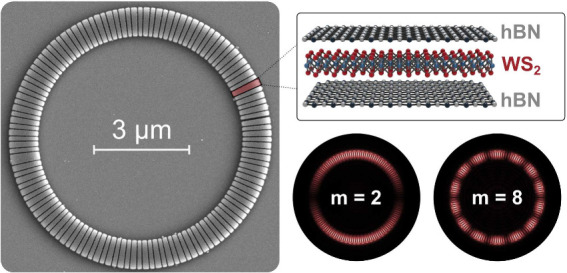

Among the diverse family of 2D materials, monolayer semiconducting TMDs such as MoS_2_, MoSe_2_, and WS_2_ stand out for strongly bound excitons with large oscillator strengths, direct band gaps in the visible to near-infrared range,^1–3^ and valley-dependent optical selection rules,^4–6^ making them ideal for exploring exciton physics and photonic device concepts.^7–9^ Coupling TMD excitons to photonic nanostructures can enhance luminescence,^10^ control nonlinear processes,^11^ and even reach strong light-matter coupling regimes.^12,13^ To harness these properties in photonic systems, 2D materials are commonly embedded in van der Waals (vdW) heterostructures, where atomically flat interfaces and low-defect dielectric environments improve optical quality. Hexagonal boron nitride (hBN) is central to this platform, providing a chemically inert and wide-bandgap (∼6 eV) encapsulation medium that reduces exciton inhomogeneous broadening and ensures stable interfaces.^14–16^

Beyond its passive role, hBN has also emerged as an active photonic material, with a low refractive index (*n* ∼ 2.1) and optical transparency across the visible and near-infrared.^17^ Patterned hBN nanostructures have enabled infrared metasurfaces that support Mie resonances, directional emission, and BICs.^18–20^ These planar arrays of nanoresonators can control phase, amplitude, and polarization of light, and when designed to support BICs, can exhibit high-*Q* resonances despite being embedded in the continuum of radiative modes.^21^ BICs arise from symmetry protection or modal interference and do not couple to the far field. By introducing controlled symmetry breaking, BICs can be transformed from dark modes to leaky modes, known as quasi-BICs (qBICs), which couple to the far field while maintaining high *Q*-factors.^22^ These qBICs have enabled advances in nonlinear optics,^23^ biosensing,^24^ and coupling to quantum emitters and 2D semiconductors.^25,26^

However, most existing qBIC metasurfaces are designed as periodic lattices with square or rectangular unit cells, which typically results in polarization sensitivity. To address these limitations, radial qBICs have been proposed as a polarization-invariant and compact alternative.^27^ Their reduced footprint and polarization robustness make them attractive for integration with monolayer TMDs, for enhanced light-matter coupling, and in applications requiring localized control of the electric field distribution, such as electro- or thermo-optic modulation.^28,29^

Rotationally symmetric nanophotonic resonators, ranging from single-resonator discs and microrings to whispering-gallery-mode structures, have been extensively studied due to their ability to confine light with high *Q*-factors and well-defined angular momentum.^30,31^ More recently, this framework has been extended through lattice-level perturbations and nanoscale unit-cell engineering, giving rise to platforms that combine global angular modes with additional photonic resonances, originating from the engineered nanoscale unit cell. Representative implementations include asymmetric microrings, radial metasurfaces, and circular photonic crystals.^27,32,33^

In this work, we design and realize a compact hBN-based radial vdW metasurface and demonstrate its integration with a WS_2_ monolayer. The geometry is tuned to the WS_2_ A-exciton and engineered to support a high-*Q* qBIC together with a discrete ladder of ring-resonator eigenmodes carrying orbital angular momentum (OAM). Full-wave numerical simulations show that the qBIC provides a radiative channel that couples these otherwise guided ring modes to the far field, producing periodically modulated dispersions observed in momentum space. Back-focal-plane (Fourier-space) photoluminescence (PL) reveals discrete momentum-space patterns consistent with this OAM ladder, with a clear one-to-one correspondence to observed transmittance behavior. Even though higher Q-factors have been reported in other dielectric platforms,^34,35^ the performance demonstrated here exceeds previous radial qBIC implementations. Importantly, the central advance of this platform lies in enabling structured ring resonator photonic states to couple to a monolayer TMD under linear excitation without relying on complex excitation schemes or integrated coupling architectures. Whereas in typical microring resonator platforms, whispering-gallery-like eigenmodes are accessed via evanescent in-plane coupling to bus waveguides,^31^ the qBIC-mediated radiative channel presented in this work allows discrete OAM-carrying eigenmodes to be accessed directly from the far field and imprinted onto excitonic emission. Overall, the radial architecture delivers polarization-invariant access to high-*Q* modes within an ∼9 μm lateral bounding box, with the actual patterned photonic region however forming a narrow ring of only ∼25 μm^2^, providing a practical route to compact and OAM-carrying light-matter interfaces in 2D materials. Potential applications include structured light-exciton emission, near-field beam shaping, and integration with tunable or active vdW photonic elements.

## Results and Discussion

### Optimization of Radial vdW Metasurfaces from hBN

Conceptually, radial metasurfaces are constructed by extracting a single row of resonators from the 2D array which constitutes a planar BIC metasurface and bending it into a circular geometry along the inter–unit-cell coupling direction, i.e., the short axis of the resonators.^36^ This transforms the 1D resonator chain into a ring-shaped structure while preserving the coupling orientation tangentially along the circumference. The radial qBIC metasurface consists of *N* = 68 double-element unit cells (136 resonators in total) arranged in a circular geometry with a radius on the order of *R* ≈ 3.95 μm, fabricated from hBN on a SiO_2_ substrate (Figure 1a). The resonators are separated by gaps on the order of 60–70 nm and feature bar lengths around 900–950 nm, with a typical hBN thickness of approximately 150–170 nm. Notably, the overall resonator dimensions could be further reduced by employing higher-index materials, owing to the comparatively lower refractive index of hBN (see Supplementary Note 1 for a detailed discussion). Exact geometrical parameters for all investigated configurations are provided in Supplementary Note 2. Previous implementations of radial qBIC structures relied on simple rectangular (rod-type) resonators with fixed dimensions, directly adapted from such 1D chains. However, while a linear chain maintains constant spacing, bending it into a ring naturally introduces a radially increasing gap between resonators, leading to nonideal coupling. We introduce a geometric improvement by transitioning from rod-type to trapezoid-type unit cells (Figure 1b). This modification restores the constant gap between resonators and increases the resonator volume compared to fixed-width designs, providing additional modal confinement in the low-index hBN platform operating close to the grating-mode cutoff. Importantly, this approach increases the effective resonator volume without necessitating thicker flakes or high-aspect-ratio geometries, which would otherwise be required to achieve comparable confinement strength in a *n* ≈ 2.1 material system. In the trapezoid configuration, the resonator width is no longer fixed, but varies continuously across the radial position. Each resonator is characterized by inner and outer widths (w_1_ and w_2_) that are calculated based on the optimized parameter set (see Supplementary Note 3). The trapezoid geometry leads to enhanced *Q*-factors, as evidenced by the approximate 20% increase observed in numerical simulations when transitioning from rod to trapezoid configurations (Figure 1c), both employing the length asymmetry introduced in previous work.^27^

**Figure 1:**
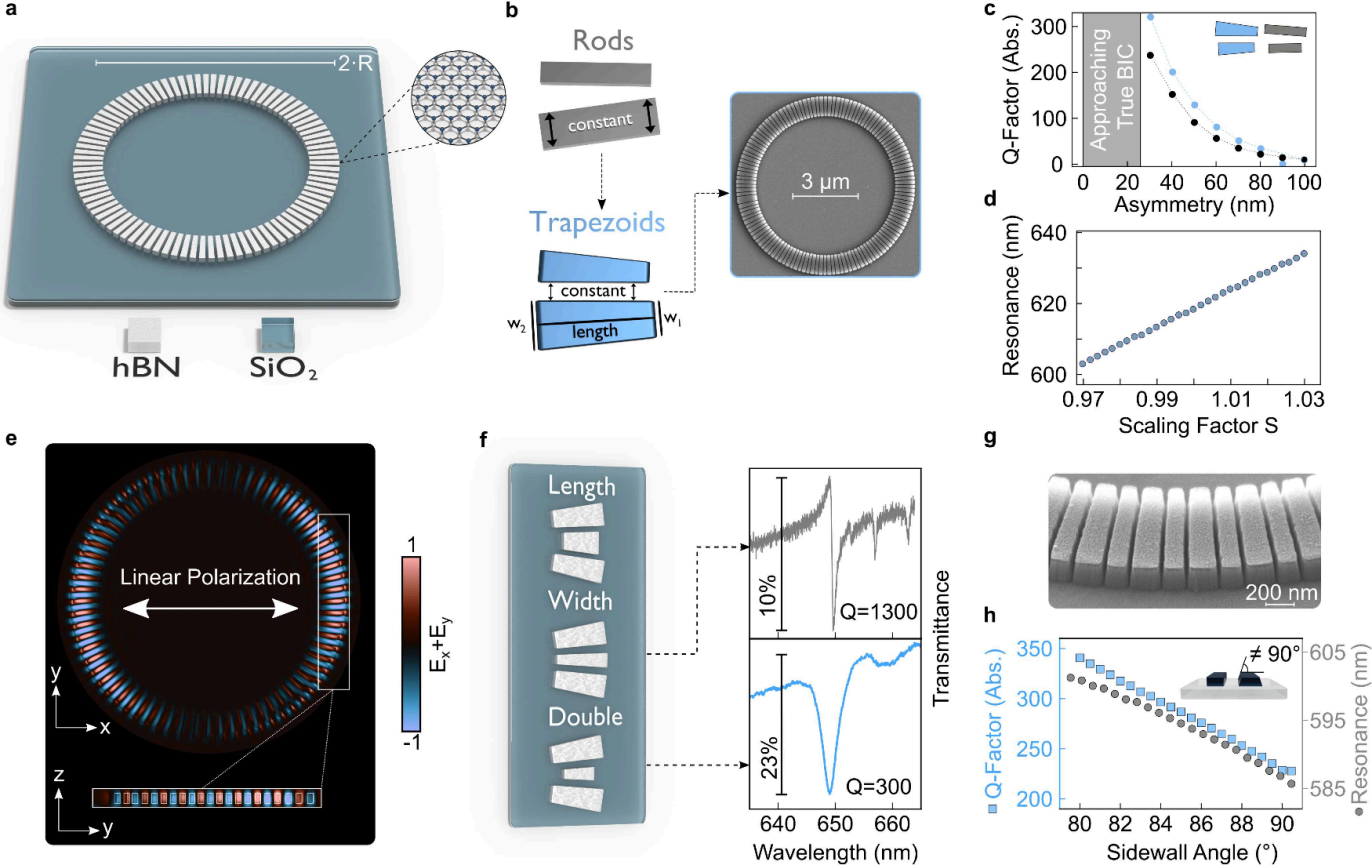
Optimization of radial qBICs in hBN. (a) The hBN crystal structure, shown in the inset, provides the fundamental building block for the radial qBIC structure with diameter 2·*R* on a SiO_2_ substrate. (b) When moving from a rod-type to a trapezoid-type unit cell (the SEM image shows a fabricated trapezoidal geometry sample with width asymmetry) the fixed parameter is no longer the width of the individual resonators but the gap between them (see Supplementary Note 3), enabling a (c) 20% increase in *Q*-factor in numerical simulations. For this, length asymmetry as introduced in previous work is used.^27^ (d) The spectral position of the resonance is tuned via a scaling factor applied to all parameters other than height and number of unit cells. (e) Sum of real parts of in-plane electric fields for radial qBIC in xy- and yz-plane. (f) Comparison of different asymmetry approaches with corresponding experimental verification, allowing for both *Q*-factor and signal optimization. (g) SEM of the fabricated structure revealing slanted sidewalls. (h) Simulations demonstrating resonance tuning and *Q*-factor control through the inclusion of slanted sidewalls. The exact structural parameters of the respective radial qBIC configurations are provided in Supplementary Note 2.

To achieve spectral tunability of the resonances, we introduce a unified scaling factor *S* that is applied to all structural parameters simultaneously, except for the resonator height (set by the thickness of the hBN flake) and the number of unit cells. This preserves the relative proportions of the design while enabling controlled tuning across a broad wavelength range (Figure 1d and Supplementary Notes 4 and 5). In contrast, previous work applied scaling only to the radius,^27^ which indeed shifts the resonance but at the cost of altering the relative geometries. The in-plane electric field distribution of the radial qBIC under linearly polarized excitation reveals the fundamental coupling mechanism within the structure (Figure 1e). The field map shows antiparallel dipoles formed in adjacent unit cells, which couple throughout the entire ring structure. The field enhancement is strongest in regions parallel or nearly parallel to the incident polarization direction, yet still appearing throughout the full structure. Due to this radial symmetry, rotating either the structure or the polarization direction produces equivalent responses, demonstrating the polarization-independent nature of the radial qBIC geometry. Previous approaches for qBIC generation in radial structures relied solely on length asymmetry to break the symmetry and achieve finite *Q*-factors (of approximately 300) in the visible spectral range.^27^ However, this approach inherently reduces the effective area of mode confinement, limiting the achievable performance. We introduce two further asymmetry control strategies that address this limitation (Figure 1f). The asymmetries are defined as relative values compared to the unperturbed symmetric system (see Supplementary 3). First, we implement width asymmetry as an alternative to length modulation. This approach achieves sizable *Q*-factors of *Q* = 1300, as demonstrated in the experimental transmittance spectrum obtained with 30% width asymmetry (Figure 1f). Second, we develop a double asymmetry approach that combines both length and width variations. The double asymmetry spectrum shown combines the 30% width asymmetry with an additional 20% length asymmetry. This technique enables higher overall asymmetries without making individual resonators too thin, to maintain structural stability, or too short, to preserve adequate mode confinement area. The doubly asymmetric configuration achieves a higher transmission modulation at the expense of a reduced *Q*-factor (*Q* = 300, Figure 1f). The reduced Q-factor reflects stronger radiative losses induced by the higher asymmetry. While higher-Q modes store energy longer, they are more sensitive to parasitic losses, such as material absorption and fabrication imperfections, which can diminish the measurable modulation depth. This flexibility allows the system to be tailored depending on whether maximum *Q*-factor or optimal signal modulation is prioritized for specific applications. The heterostructure discussed in later sections employs this double-asymmetry. Beyond the in-plane geometric optimizations, we introduce an additional out-of-plane tuning mechanism through controlled sidewall etching angles (Figure 1g and h). This approach addresses fundamental fabrication limitations by circumventing minimum achievable gap sizes through slanted sidewall profiles. The anisotropic etching not only provides an alternative pathway to reduce effective gap dimensions but also serves as a powerful tool for both *Q*-factor enhancement and spectral tuning. The relationship between sidewall angle and optical performance is demonstrated through simulated characterization, showing substantial *Q*-factor improvements concurrent with resonance wavelength shifts. Throughout this work, a sidewall angle of 85° is employed, representing an optimal compromise between fabrication reliability and optical performance.

### Radial qBIC-Mediated Coupling of Ring Resonator Modes to the Far Field

The angular response of radial qBICs reveals a rich interplay between localized unit-cell resonances and collective ring eigenmodes. To systematically study this behavior, we define the incident polarization states relative to the angled excitation geometry (Figure 2a and b). For oblique incidence, TE-mode excitation corresponds to the electric field oriented perpendicular to the plane of incidence, while TM-mode maintains the electric field within the incidence plane. This distinction becomes crucial when analyzing the angle-dependent transmittance spectrum. Under TE-mode excitation, the angle-resolved transmission spectrum exhibits a periodically modulated parabolic profile (Figure 2c). While the underlying parabolic envelope is consistent with previous demonstrations of qBIC behavior,^37,38^ the varying modulation represents a feature arising from the specific geometry and excitation conditions of our radial qBIC system. This behavior becomes even more pronounced under TM-mode excitation (Figure 2d), where the dispersion reveals not only the periodically modulated parabolic envelope but multiple distinct parabolic branches. This multibranch structure indicates the presence of several modes that contribute to the overall optical response, suggesting a rich landscape of different electromagnetic eigenmodes in the system.

**Figure 2:**
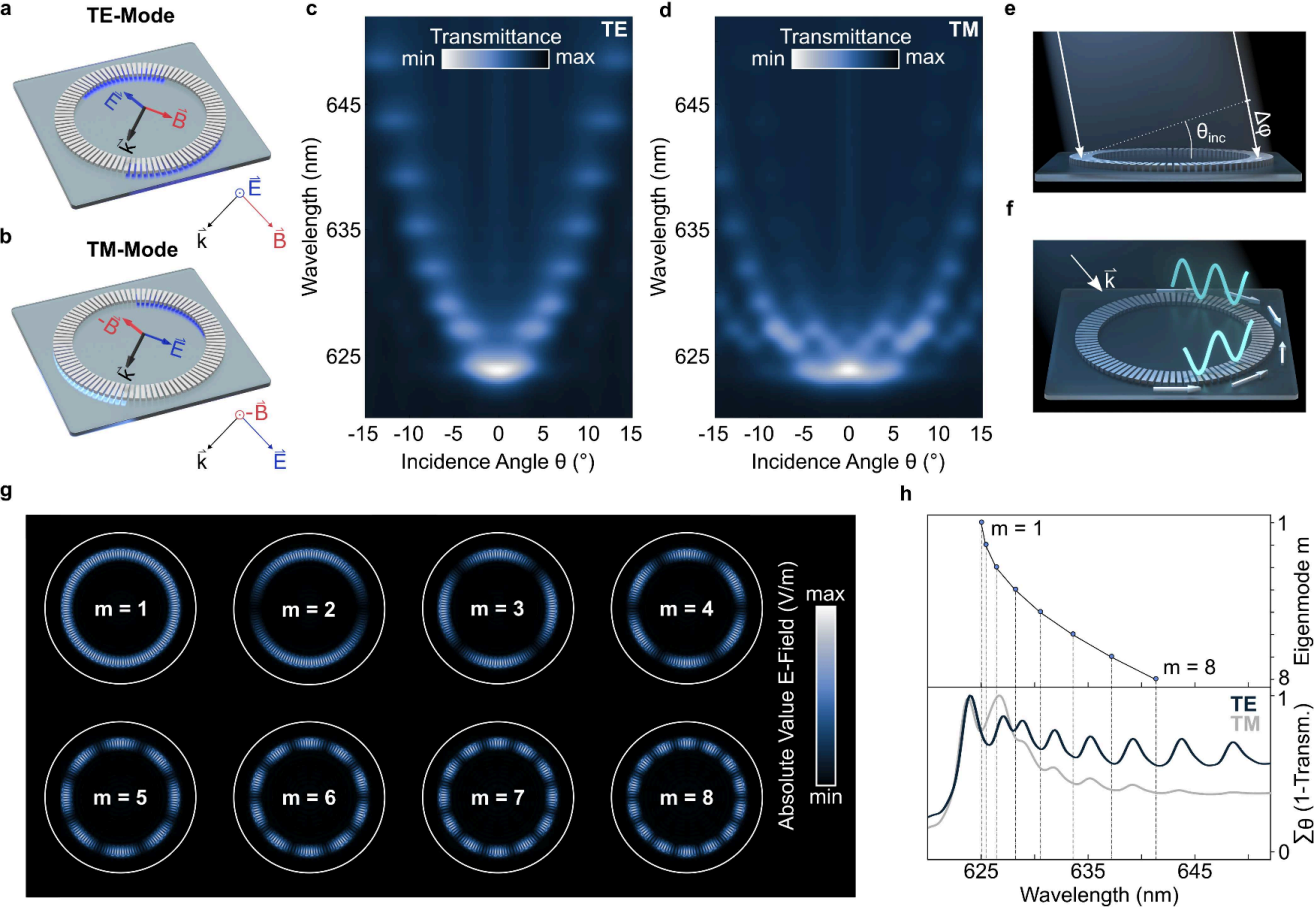
Radial qBIC in k-space. (a, b) Sketch of the constituent TE and TM mode fields of the radial qBIC structure under oblique incidence. (c) Simulated transmittance dependency for oblique incidence in TE-mode. The resonance trend exhibits a periodically modulated parabolic shape. (d) Simulated transmittance dependency for oblique incidence in TM-mode. In this case, the resonance trend exhibits a shape constituted by a series of periodically modulated parabolas. (e) Sketch of the resulting phase difference *Δφ* at opposing sides of the radial qBIC structure. (f) Sketch of angled light, launching propagating waves at the respective ring poles with opposing propagation direction, resulting in a standing wave. (g) Absolute value of the electric field for the first eight ring eigenmodes (mode number 1 to 8) of the radial qBIC structure. (h) Spectral position of the respective eigenmodes correlated with (1 – T) summed up over all angles of incidence for both TE and TM. The exact structural parameters of the respective radial qBIC configurations are provided in Supplementary Note 2.

The origin of the multiple resonance branches can be understood through a phase accumulation mechanism that occurs during oblique incidence (Figure 2e). When the excitation angle deviates from normal incidence, phase differences accumulate across the structure. In TM-mode, this phase accumulation occurs in the electric field component that directly drives the resonant response through electric dipole coupling. Consequently, different phase accumulation conditions create multiple solutions that manifest as distinct resonance branches in the angular dispersion. The periodically modulated features arise from ring resonator eigenmodes excited under tilted illumination, as illustrated schematically in Figure 2f: angled excitation launches counter-propagating waves at opposite ring poles, whose interference produces the standing-wave patterns that define the eigenmodes around the ring. Although the excitation angle is continuous, the ring geometry supports azimuthal eigenmodes. As a result, only specific in-plane momenta efficiently couple to the structure, leading to the observed discrete branch structure. In conventional microring platforms, such whispering-gallery or OAM eigenmodes are guided resonances, confined by total internal reflection and typically accessed only via evanescent in-plane coupling schemes such as bus waveguides or tapered fibers.^39^ The large in-plane wavevector of such circulating modes typically cannot couple directly to free-space plane waves, making them effectively dark to far-field excitation and detection. In our radial qBIC system, however, the leaky qBIC resonance provides a built-in radiative channel, thereby mediating coupling between the normally bound OAM states and free-space radiation. As a result, modes that would remain hidden in standard microrings become directly observable in angle-resolved transmittance and PL. This mechanism effectively transforms the radial qBIC into a far-field interface for the OAM ladder of ring eigenmodes, enabling their study without the need for waveguide coupling or near-field probes.

To substantiate our interpretation of the periodically modulated envelope as ring eigenmodes made radiatively accessible by the qBIC, we compute the first eight eigenmodes of the radial structure using COMSOL (Figure 2g). Each solution is characterized by a well-defined OAM l=m , in line with prior reports on photonic crystal microrings.^32^ These solutions (*m* = 2, 3, 4, ..., 8) represent distinct OAM states that are normally confined by total internal reflection but become radiatively coupled through the qBIC. Their characteristic *m*-fold azimuthal field patterns directly reflect their angular momentum content.

The correspondence between these eigenmodes and the transmittance behavior of the same structure is demonstrated in Figure 2h: the eigenfrequencies calculated for the ring resonator align closely with the oscillatory features in the angle-resolved calculated (1 – transmittance) spectra of the same structure. This spectral agreement confirms that the features observed in the angular dispersion indeed arise from a ladder of OAM-carrying eigenmodes. Rather than enhancing only a single resonance, the radial qBIC opens a radiative access channel to these guided modes, which would otherwise remain confined to the near field unless additional outcoupling structures, such as gratings, were introduced.

### Radial qBIC vdW-Heterostructure

To explore exciton-photon interactions in our system, we fabricated a vdW heterostructure consisting of a WS_2_ monolayer sandwiched between two 80 nm thick hBN flakes, and subsequently patterned it into the radial qBIC platform (Figure 3a–e). The WS_2_ monolayer has a direct bandgap and strong excitonic response at room temperature, resulting in bright room-temperature PL emission (Figure 3d). The encapsulation in hBN furthermore provides an atomically flat environment that preserves optical quality and minimizes inhomogeneous broadening.^37^ To facilitate the interaction between the radial qBIC and the WS_2_ exciton, we fine-tuned the structure’s geometry to place the qBIC resonance near 620 nm, spectrally aligning it with the exciton energy. This alignment establishes the conditions under which exciton-photon interaction may be enhanced, potentially entering the strong coupling regime. To probe the polarization symmetry of the qBIC-enhanced emission, we image the PL intensity at the exciton wavelength (λ = 620 nm) under fixed linear polarization and repeat the measurement after rotating the sample by 90°. Because the radial qBIC is isotropic, this is equivalent to rotating the polarization relative to the structure. For clarity, this is depicted as a polarization rotation in Figure 3g. The resulting PL maps remain nearly unchanged up to rotation, in line with the polarization-invariant response of the radial qBIC. The PL intensity is enhanced in regions of the structure where the long axis of the resonators is parallel to the excitation, corresponding to the areas with the strongest near fields (Figure 1g), which suggests local field concentration and resonant enhancement of excitonic emission. Spectral measurements further indicate enhanced light-matter interaction. The normalized PL and transmittance spectra for a structure scaled to *S* = 1.01, show a clear spectral overlap between the PL peak and the radial qBIC resonance (Figure 3f). This alignment indicates that the qBIC may enhance exciton emission by increasing the local density of optical states and facilitating efficient radiative coupling. Under the right conditions radial qBIC can generate self-hybridized exciton-polaritons, opening the door for future experimental studies of strong coupling in such radially symmetric systems (see Supplementary Note 6).

**Figure 3:**
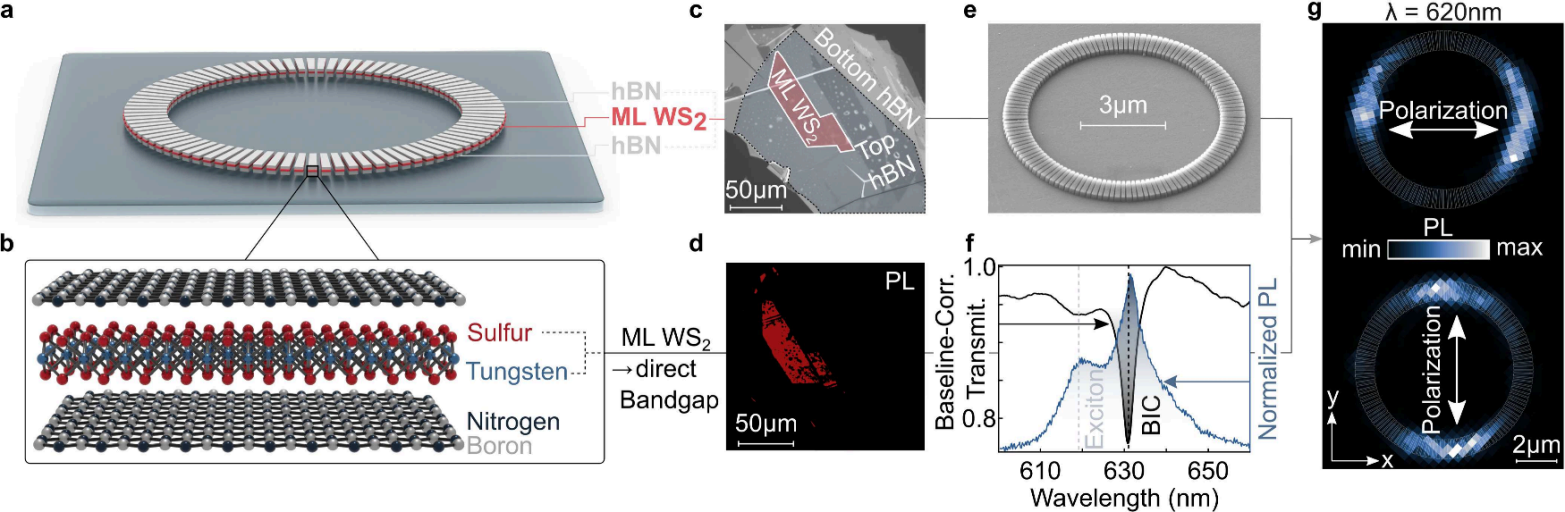
Radial qBIC vdW-heterostructure. (a) Radial qBIC vdW-heterostructure with a monolayer (ML) of WS_2_ encapsulated by two bulk layers of hBN. (b) Respective lattice structures of heterostructure components. (c) Optical microscope image of final heterostructure. Both the bottom and top layer of hBN are 80 nm thick. (d) PL microscope image of the unpatterned heterostructure shows signal from the area of the monolayer. (e) SEM image of the fabricated structure. (f) Baseline-corrected transmittance and normalized PL spectra for radial qBIC with scaling factor *S* = 1.0075. Both the excitonic response and a BIC-driven resonant enhancement are visible in the PL spectrum. For reference the PL from the corresponding unpatterned heterostructure, see ref 37. (g) Experimental PL maps of radial qBIC vdW-heterostructure close to the excitonic wavelength (λ = 620 nm) show BIC-driven enhancement of PL emission. The exact structural parameters of the respective radial qBIC configurations are provided in Supplementary Note 2.

### k-Space Hyperspectral Imaging of Radial qBIC vdW-Heterostructure

Figures 4a and 4b display hyperspectral *k*-space PL images for TE and TM polarizations, respectively. In both polarizations, multiple dispersive features are visible, forming parabolic ripple patterns consistent with eigenmode simulations (Figure 2g). For TE (Figure 4a), the emission is dominated by a single parabolic branch, whereas in TM (Figure 4b) several higher-order contributions appear. To connect this emission to the linear optical response, we integrate the PL intensity over all emission angles, resulting in an angle-integrated PL spectrum. This is directly compared to the real-space PL spectrum collected from the entire radial qBIC of the same structure (Figure 4c). A clear correspondence emerges between the resonance peaks in the PL and the momentum resolved features. Reciprocity-based simulations^40^ reproduce the observed PL dispersions, confirming that the PL emission reflects the underlying WGM-like eigenmodes of the radial qBIC structure (Supplementary Note 7). To evaluate the robustness of this behavior under varying excitation conditions, we measure the PL spectrum as a function of pump power (Figure 4d). Even at the lowest excitation fluences, the ripple structure remains clearly discernible, indicating that the underlying mode coupling is governed primarily by the geometry and remains stable. As the excitation power increases by 3 orders of magnitude, these ripple features persist across the spectrum, demonstrating the resilience of the hybrid modes under higher carrier densities and elevated local fields. Fitting the peak positions of selected modes (labeled I, II, and III in Figure 4c), we observe a subtle but systematic blueshift in emission energy with increasing pump power (Figure 4e). This shift is modest, on the order of a few meV, but reproducible, and may be attributed to weak band-filling effects or changes in exciton binding energy due to photoinduced screening in the monolayer.^41^ Another possible explanation could be light-induced renormalization of excitonic transitions, a known phenomenon in monolayer TMDs under elevated carrier densities.^42^ Importantly, the linewidths of the hybrid peaks show no dramatic broadening with increasing power, indicating that the system does not enter a loss-dominated or saturated regime within the explored power range. Overall, these results are consistent with momentum-resolved coupling between excitonic emission from the monolayer and the structured photonic modes supported by the radial qBIC platform, with both spectral and angular features showing stability across a broad range of excitation conditions. Notably, the ability to recover the characteristic patterns associated with OAM modes in the PL response suggests that these angular features persist in the presence of exciton-photon interaction. This indicates that the underlying OAM structure of the qBIC modes may be partially imprinted onto the excitonic emission.

**Figure 4:**
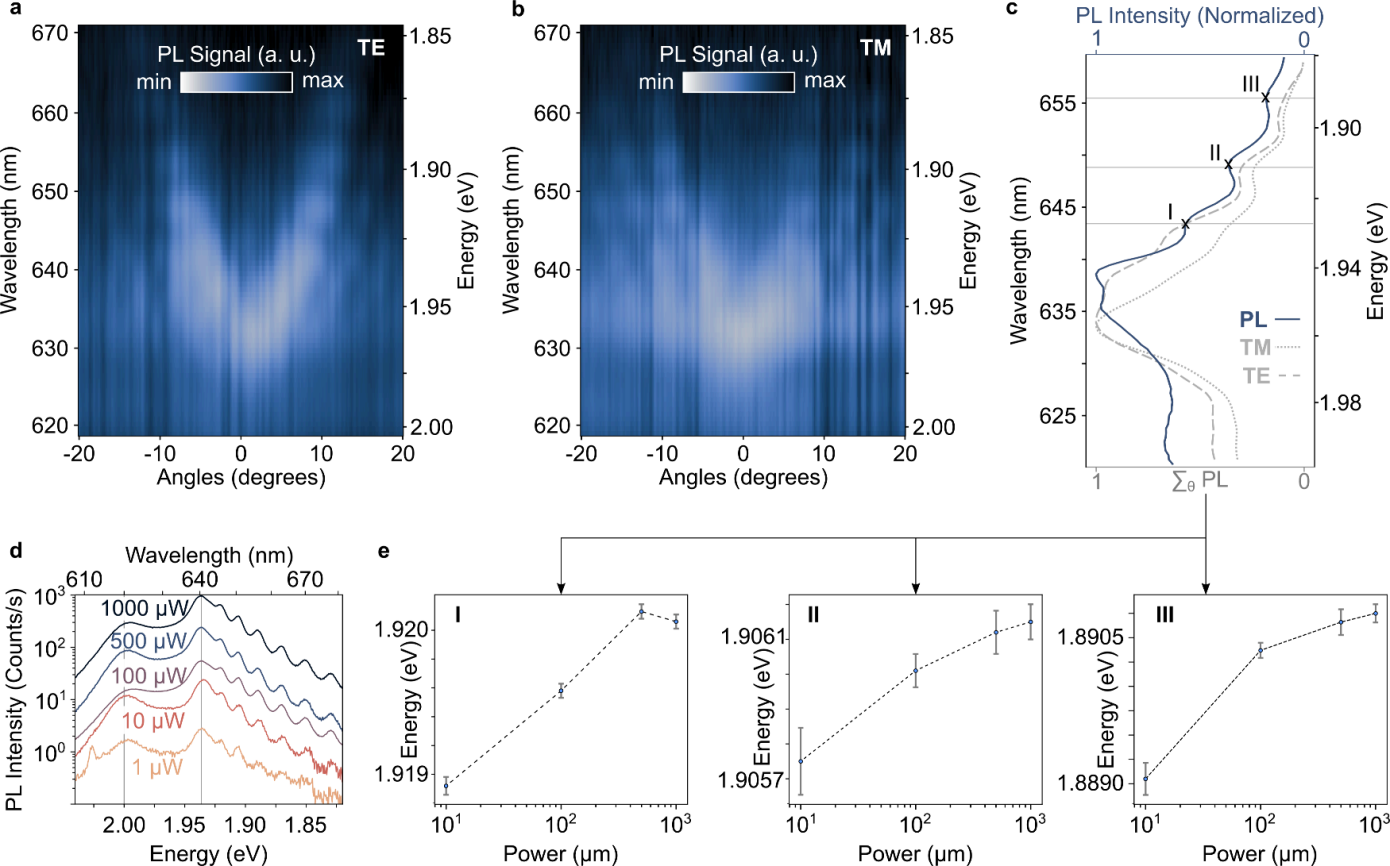
k-Space hyperspectral imaging of radial qBIC vdW-heterostructure. Vertical cross-section of the experimental 3D hyperspectral image showing a periodically modulated parabolic dispersion for (a) TE and (b) TM. Low-mode number modes near the qBIC resonance are not distinctly resolved in the experiment. (c) Experimental real-space PL spectrum collected from the radial qBIC compared with the experimental k-space-resolved PL summed over all in-plane wave vectors for TE and TM excitation for the same structure. (d) Power-dependent PL of radial qBIC vdW-heterostructure. (e) Power-dependence of the peak-position and linewidth of three separate local maxima in the PL spectrum. The exact structural parameters of the respective radial qBIC configurations are provided in Supplementary Note 2.

## Conclusion

We employ a periodic, rotationally symmetric architecture with a nanoscale perturbation motif, enabling discrete ring eigenmodes, symmetry-protected qBICs, and enhanced light-matter interactions. While conceptually derived from microring resonators, our implementation adopts a fully metasurface-based design that combines global symmetry with local control to enable compact and tunable optical functionality.

Importantly, the radial architecture enables a combination of properties not simultaneously accessible in conventional grating or photonic crystal slab geometries, namely polarization invariance and discrete OAM-carrying eigenmodes originating from the underlying ring geometry as well radiative mediation via a qBIC resonance. Furthermore, whispering-gallery-like ring modes are directly accessible under linear far-field excitation, without requiring evanescent in-plane coupling or complex excitation schemes.

Compared to earlier radial qBIC demonstrations,^27^ our structures exhibit markedly improved performance: for the same *Q*-factor, we achieve over 2.5× greater spectral modulation, and for fixed modulation depth, more than a 4-fold increase in *Q*-factor, even in a low-index system based on hBN (previous work used silicon). Although this comes with a modest increase in device footprint relative to high-index implementations, our structures remain more compact than typical hBN metasurface approaches. Initial hBN-based qBIC designs required patterned areas exceeding 400 μm^2^,^20^ whereas our radial qBICs are confined to a circular footprint with an outer radius of ∼4.5 μm (area ∼64 μm^2^), corresponding to less than one-sixth of the previously reported platform size. Importantly, the photonically active region consists only of a narrow annulus of approximately 1 μm width, as the central area remains unpatterned. The actual patterned area is therefore ∼25 μm^2^, i.e., roughly one-16th of the previously reported implementations. This unpatterned central region can be leveraged for spectral multiplexing, emitter integration, or additional photonic functionality, providing further design flexibility without increasing the overall device footprint. Importantly, these advantages are not accompanied by a drop in performance; our optimized radial geometry reaches *Q*-factors up to 1300, nearly four times higher than the ∼300 reported for the initial periodic resonator lattice hBN designs,^20^ underscoring the efficiency of this approach in shaping and confining optical modes. Our results also demonstrate that integrating monolayer WS_2_ into the radial qBIC architecture enables clear signatures of light-matter interaction between the excitonic and photonic components. While direct experimental evidence of strong coupling has not yet been achieved, the spectral overlap between PL and the qBIC resonance, together with momentum-resolved PL, suggests enhanced coupling and structured emission. The persistence of ripple-like features across excitation powers, including weak but reproducible power-dependent shifts, supports the view that the photonic mode structure plays an active role in shaping the excitonic response. Ring eigenmode hybridization has previously been explored in asymmetric microring photonic crystals,^32^ where discrete features were confirmed via near- and far-field intensity measurements. However, those studies focused on purely photonic modes outside the visible range and did not investigate excitonic interaction. The reappearance of ring eigenmodes, known to carry OAM, in the PL indicates that OAM content is at least partially transferred to, or preserved within, the qBIC-exciton-coupled emission. This points to the possibility of imprinting angular momentum structure onto light generated from 2D materials via nanophotonic symmetry engineering. As detailed in Supporting Note 8, we further demonstrate that these ring eigenmodes are not merely passive signatures but can be actively controlled via ultrafast, polarization-selective refractive index modulation and selectively addressed using OAM-carrying structured light, establishing dynamic momentum-space reconfiguration and mode-selective excitation as practical extensions of the radial qBIC concept. Such momentum-structured emission could provide a foundation for more complex spatial and spectral control. In particular, the ring-like, intensity-modulated PL distributions may enable exciton guidance, localization, or spatially varying light-matter coupling within a single device. These effects open potential pathways for exciton trapping or valley-selective routing, where spin, valley, and momentum degrees of freedom could be coherently manipulated. Additionally, the PL far-field response carrying OAM may enable on-chip information encoding.

## Methods

### Numerical Simulations

Simulations of the transmittance spectra for the radial-BIC hBN metasurfaces were conducted using the finite-difference time-domain (FDTD) package of a commercial software (Lumerical Ansys). The refractive index of the SiO_2_ substrate was set to 1.45, while that of hBN was taken from literature.^27^ The full structure was simulated using perfectly matched layer (PML) boundary conditions. The eigenmodes of the ring were calculated with the eigenfrequency solver of the wave optics module in COMSOL Multiphysics. Here, the refractive index of hBN was assumed to be a constant value of *n* = 2.12, and the entire ring was considered embedded in air. Due to symmetry, only one-half of the ring was simulated, with perfect magnetic conductor (PMC) boundary conditions applied at the symmetry plane.

### Sample Fabrication

Fused silica substrates were initially cleaned by sonication in acetone at 55 °C, followed by isopropanol to remove any residual acetone. Subsequently, the substrates were treated with O_2_ plasma to eliminate organic residue and enhance flake adhesion. To facilitate precise global alignment of the flake position on the substrate during subsequent processing, a marker system was created on the substrates using optical lithography (SÜSS Maskaligner MA6). The hBN flakes were mechanically exfoliated from bulk crystals (HQ Graphene) onto the cleaned silica marker substrates. The deposition process was conducted at a temperature of 105 °C to evaporate moisture and stretch the exfoliation tape, ensuring flattened transferred flakes. The height of the flakes was measured using a profilometer (Bruker Dektak XT) with a stylus having a radius of 2 μm. The radial hBN metasurfaces were fabricated using an electron-beam lithography (EBL) process, followed by lift-off and reactive-ion etching (RIE). The EBL step was carried out using an eLINE Plus (Raith Nanofabrication) with 20 kV acceleration voltage and a 10 μm aperture. A single layer of positive-tone AR-P 6200.13 (Allresist) was used as the EBL resist, spin-coated at 1200 rpm and baked at 175 °C for 5 min. Espacer 300Z (Showa Denko K.K.) was then subsequently spin-coated onto the sample. The patterned films were developed in amyl acetate, followed by a mixture of methyl isobutyl ketone and isopropyl alcohol (1:9 ratio). A hardmask consisting of 2 nm titanium (Ti) and 35 nm of chromium (Cr) was evaporated onto the sample using electron-beam evaporation and subsequently lifted off overnight in Microposit Remover 1165 (Microresist). This served as an etching mask for the subsequent reactive-ion etching process, using sulfur hexafluoride (SF_6_) and argon (Ar) gases under 6.0 mTorr pressure with 300 W HF and 150 W ICP power. The Cr part of the hardmask was removed via reactive-ion etching based on chlorine (Cl_2_) and oxygen (O_2_) at a pressure of 12 mTorr with 20 W HF power and 500 W ICP power. The Ti part of the hardmask was subsequently removed using a solution of potassium monoiodide and iodine (Sigma-Aldrich). The full fabrication process is depicted in Supplementary Note 9.

### Linear Optical Measurements

The linear transmittance spectra were characterized using a commercially available confocal optical transmittance microscope (Witec alpha 300 series). All experimental transmittance spectra were referenced to the bare SiO_2_ substrate unless stated otherwise. The samples were illuminated from the bottom using collimated and linearly polarized white light from a broadband halogen lamp (Thorlabs OSL2). The light was subsequently confocally collected with a 50× objective (NA = 0.8) and coupled into a multimode fiber. The collected signal was guided into a spectrometer with a grating groove density of 600 grooves/mm, where it was dispersed onto a Si-CCD sensor.

### k-Space Hyperspectral Microscope

To perform the k-space hyperspectral PL measurements, we employed a custom hyperspectral microscopy setup (a sketch of this setup is depicted in Supplementary Note 10). The excitation beam is produced by a 532 nm continuous-wave (CW) laser, coupled to a multimode fiber (core diameter: 100 μm), whose tip is imaged onto the sample using a collimation lens (not shown). The laser beam is reflected by a dichroic mirror (RazorEdge 532 nm) toward a 100× objective (NA = 0.75), which is also used for signal collection. The PL emitted by the sample passes through the dichroic mirror and a long-pass filter (LP550), used to further suppress residual excitation light. A Fourier lens in the detection path enables imaging of the back focal plane of the objective onto the camera. Before detection, the Translating-Wedge-Based Identical Pulses eNcoding System (TWINS) interferometer is inserted in the optical path.^43^ By varying the delay between the two replicas of the Fourier-space image generated by the TWINS interferometer, we acquire an interferogram for each pixel. The Fourier transform of each interferogram yields the corresponding PL spectrum. The final data set is a three-dimensional datacube (hypercube) of PL intensity as a function of θ_*x*_, θ_*y*_, and energy. Vertical cross sections of this hypercube reveal the photonic mode dispersion of the sample for different polarizations (see main text), enabled by the built-in polarizers of the TWINS interferometer. These are set at 45° to select the TE (TM) mode along the diagonal (antidiagonal), respectively.^44^

## Supplementary Material



## Data Availability

All data needed to evaluate the conclusions in the paper are present in the paper and/or the Supporting Information.

## Notes

Si to hBN transition, list of structural parameters, definitions trapezoidal unit cell and relative asymmetry, impact of geometric parameters, scaling factor, numerical investigation of strong coupling, reciprocity-based simulations of photoluminescence spectra, optical manipulation, illustration fabrication workflow, sketch experimental setup (PDF)

## References

[1] Mak K. F., Lee C., Hone J., Shan J., Heinz T. F. (2010). Atomically thin
MoS 2: a new direct-gap semiconductor. Phys.
Rev. Lett..

[2] Wang G., Chernikov A., Glazov M. M., Heinz T. F., Marie X., Amand T., Urbaszek B. (2018). Colloquium: Excitons in atomically
thin transition metal dichalcogenides. Rev.
Mod. Phys..

[3] Chernikov A., Berkelbach T. C., Hill H. M., Rigosi A., Li Y., Aslan B., Reichman D. R., Hybertsen M. S., Heinz T. F. (2014). Exciton binding energy and nonhydrogenic Rydberg series
in monolayer WS 2. Phys. Rev. Lett..

[4] Xiao D., Liu G.-B., Feng W., Xu X., Yao W. (2012). Coupled spin
and valley physics in monolayers of MoS 2 and other group-VI dichalcogenides. Phys. Rev. Lett..

[5] Ye Z., Cao T., O’brien K., Zhu H., Yin X., Wang Y., Louie S. G., Zhang X. (2014). Probing excitonic
dark
states in single-layer tungsten disulphide. Nature.

[6] Dufferwiel S. (2015). Exciton-polaritons
in van der Waals heterostructures embedded in
tunable microcavities. Nat. Commun..

[7] Novoselov K. S., Mishchenko A., Carvalho A., Castro Neto A. H. (2016). 2D materials
and van der Waals heterostructures. Science.

[8] Geim A. K., Grigorieva I. V. (2013). Van der
Waals heterostructures. Nature.

[9] Bonaccorso F., Sun Z., Hasan T., Ferrari A. C. (2010). Graphene photonics and optoelectronics. Nat. Photonics.

[10] Li C., Luo H., Hou L., Wang Q., Liu K., Gan X., Zhao J., Xiao F. (2024). Giant photoluminescence enhancement
of monolayer WSe2 using a plasmonic nanocavity with on-demand resonance. Nano Lett..

[11] Seyler K. L., Schaibley J. R., Gong P., Rivera P., Jones A. M., Wu S., Yan J., Mandrus D. G., Yao W., Xu X. (2015). Electrical
control of second-harmonic generation in a WSe2 monolayer transistor. Nat. Nanotechnol..

[12] Flatten L. C., He Z., Coles D. M., Trichet A. A., Powell A. W., Taylor R. A., Warner J. H., Smith J. M. (2016). Room-temperature exciton-polaritons
with two-dimensional WS2. Sci. Rep..

[13] Lundt N., Klembt S., Cherotchenko E., Betzold S., Iff O., Nalitov A. V., Klaas M., Dietrich C. P., Kavokin A. V., Höfling S., Schneider C. (2016). Room-temperature Tamm-plasmon exciton-polaritons
with a WSe2 monolayer. Nat. Commun..

[14] Dean C. R., Young A. F., Meric I., Lee C., Wang L., Sorgenfrei S., Watanabe K., Taniguchi T., Kim P., Shepard K. L., Hone J. (2010). Boron nitride substrates for high-quality
graphene electronics. Nat. Nanotechnol..

[15] Wang L., Meric I., Huang P., Gao Q., Gao Y., Tran H., Taniguchi T., Watanabe K., Campos L., Muller D., Guo J., Kim P., Hone J., Shepard K. L., Dean C. R. (2013). One-dimensional
electrical contact
to a two-dimensional material. Science.

[16] Ajayi O. A., Ardelean J. V., Shepard G. D., Wang J., Antony A., Taniguchi T., Watanabe K., Heinz T. F., Strauf S., Zhu X.-Y., Hone C. (2017). Approaching the intrinsic photoluminescence
linewidth in transition metal dichalcogenide monolayers. 2D Materials.

[17] Biechteler J., Heimig C., Weber T., Gryb D., Sortino L., Maier S. A., de S. Menezes L., Tittl A. (2025). Fabrication Optimization
of van der Waals Metasurfaces: Inverse Patterning Boosts Resonance
Quality Factor. Advanced Optical Materials.

[18] Giles A. J., Dai S., Vurgaftman I., Hoffman T., Liu S., Lindsay L., Ellis C. T., Assefa N., Chatzakis I., Reinecke T. L., Tischler J. G., Fogler M. M., Edgar J. H., Basov D. N., Caldwell J. D. (2018). Ultralow-loss
polaritons in isotopically
pure boron nitride. Nat. Mater..

[19] Autore M., Li P., Dolado I., Alfaro-Mozaz F. J, Esteban R., Atxabal A., Casanova F., Hueso L. E, Alonso-Gonzalez P., Aizpurua J., Nikitin A. Y, Velez S., Hillenbrand R. (2018). Boron nitride
nanoresonators for phonon-enhanced molecular vibrational spectroscopy
at the strong coupling limit. Light: Science
& Applications.

[20] Kühner L., Sortino L., Tilmann B., Weber T., Watanabe K., Taniguchi T., Maier S. A., Tittl A. (2023). High-Q nanophotonics
over the full visible spectrum enabled by hexagonal boron nitride
metasurfaces. Adv. Mater..

[21] Hsu C. W., Zhen B., Stone A. D., Joannopoulos J. D., Soljačić M. (2016). Bound states in the
continuum. Nature Reviews Materials.

[22] Koshelev K., Lepeshov S., Liu M., Bogdanov A., Kivshar Y. (2018). Asymmetric
metasurfaces with high-Q resonances governed by bound states in the
continuum. Phys. Rev. Lett..

[23] Koshelev K., Tang Y., Li K., Choi D.-Y., Li G., Kivshar Y. (2019). Nonlinear metasurfaces
governed by bound states in
the continuum. ACS Photonics.

[24] Tittl A., Leitis A., Liu M., Yesilkoy F., Choi D.-Y., Neshev D. N., Kivshar Y. S., Altug H. (2018). Imaging-based molecular
barcoding with pixelated dielectric metasurfaces. Science.

[25] Do T. T. H., Nonahal M., Li C., Valuckas V., Tan H. H., Kuznetsov A. I., Nguyen H. S., Aharonovich I., Ha S. T. (2024). Room-temperature
strong coupling in a single-photon emitter-metasurface
system. Nat. Commun..

[26] Al-Ani I. A., As’ Ham K., Huang L., Miroshnichenko A. E., Hattori H. T. (2021). Enhanced strong coupling of TMDC monolayers by bound
state in the continuum. Laser & Photonics
Reviews.

[27] Kühner L., Sortino L., Berté R., Wang J., Ren H., Maier S. A., Kivshar Y., Tittl A. (2022). Radial bound states
in the continuum for polarization-invariant nanophotonics. Nat. Commun..

[28] Li B., Zu S., Zhou J., Jiang Q., Du B., Shan H., Luo Y., Liu Z., Zhu X., Fang Z. (2017). Single-nanoparticle
plasmonic electro-optic modulator based on MoS2 monolayers. ACS Nano.

[29] Gan X., Englund D., Van Thourhout D., Zhao J. (2022). 2D materials-enabled
optical modulators: From visible to terahertz spectral range. Applied Physics Reviews.

[30] Little B. E., Chu S. T., Haus H. A., Foresi J., Laine J.-P. (1997). Microring
resonator channel dropping filters. Journal
of Lightwave Technology.

[31] Vahala K. J. (2003). Optical
microcavities. Nature.

[32] Wu R., Chen B., Liu D., Qiu G., Liu Z., Wei D., Liu J. (2025). Revealing Resonant
Mode Properties in Asymmetric Photonic
Crystal Microrings through Diverse Excitation Methods. Nano Lett..

[33] Ma C., Yang J., Li P., Rugeramigabo E. P., Zopf M., Ding F. (2024). Circular photonic crystal
grating
design for charge-tunable quantum light sources in the telecom C-band. Opt. Express.

[34] Kodigala A., Lepetit T., Gu Q., Bahari B., Fainman Y., Kanté B. (2017). Lasing action from photonic bound states in continuum. Nature.

[35] Chen M.-H., Xing D., Su V.-C., Lee Y.-C., Ho Y.-L., Delaunay J.-J. (2023). GaN ultraviolet laser based on bound states in the
continuum (BIC). Advanced Optical Materials.

[36] Gölz T., Baù E., Aigner A., Mancini A., Barkey M., Keilmann F., Maier S. A., Tittl A. (2024). Revealing Mode Formation
in Quasi-Bound States in the Continuum Metasurfaces via Near-Field
Optical Microscopy. Adv. Mater..

[37] Sortino L., Biechteler J., Lafeta L., Kühner L., Hartschuh A., de S. Menezes L., Maier S. A., Tittl A. (2025). Atomic-layer
assembly of ultrathin optical cavities in van der Waals heterostructure
metasurfaces. Nat. Photonics.

[38] Jiang Q., Hu P., Wang J., Han D., Zi J. (2023). General bound states
in the continuum in momentum space. Phys. Rev.
Lett..

[39] Hammer, M. ; Ebers, L. ; Förstner, J. In Complex Light and Optical Forces XVI; Andrews, D. L. , Galvez, E. J. , Rubinsztein-Dunlop, H. , Eds.; SPIE: San Francisco, United States, 2022.

[40] Maksimov A., Tartakovskii I., Filatov E., Lobanov S., Gippius N., Tikhodeev S., Schneider C., Kamp M., Maier S., Höfling S., Kulakovskii V. D. (2014). Circularly polarized light emission
from chiral spatially-structured planar semiconductor microcavities. Phys. Rev. B.

[41] Chernikov A., Ruppert C., Hill H. M., Rigosi A. F., Heinz T. F. (2015). Population
inversion and giant bandgap renormalization in atomically thin WS2
layers. Nat. Photonics.

[42] Steinhoff A., Florian M., Rösner M., Schönhoff G., Wehling T. O., Jahnke F. (2017). Exciton fission in
monolayer transition
metal dichalcogenide semiconductors. Nat. Commun..

[43] Brida D., Manzoni C., Cerullo G. (2012). Phase-locked
pulses for two-dimensional
spectroscopy by a birefringent delay line. Opt.
Lett..

[44] Genco A., Cruciano C., Corti M., McGhee K. E., Ardini B., Sortino L., Hüttenhofer L., Virgili T., Lidzey D. G., Maier S. A., Bassi A., Valentini G., Cerullo G., Manzoni C. (2022). k-Space hyperspectral imaging by
a birefringent common-path interferometer. ACS
Photonics.

