# Supplementary material for: Integration of 2D Materials in Radial van der Waals Heterostructure Metasurfaces
Source: ACS Nano. 2026 May 21;20(22):15927–36. doi: 10.1021/acsnano.5c20740 (PMC13255532; doi:10.1021/acsnano.5c20740)
Supplement: Supplementary file 1 [file nn5c20740_si_001.pdf]

Supporting Information for Integration of 2D Materials in Radial van der Waals Heterostructure Metasurfaces

Connor Heimig[1], Jonas Biechteler[1], Cristina Cruciano[2], Armando Genco[2,3], Thomas Weber[1], Michael Hirler[1], Dmytro Gryb[1], Leonardo de S. Menezes[1,4], Gianluca Valentini[2,5], Cristian Manzoni[5], Giulio Cerullo[2,5], Stefan A. Maier[6,7], Alexander A. Antonov[*1], Luca Sortino[†1], and Andreas Tittl[‡1,8]

[1]Chair in Hybrid Nanosystems, Nanoinstitute Munich, Faculty of Physics, Ludwig-Maximilians-University, 80539 Munich, Germany
[2]Dipartimento di Fisica, Politecnico di Milano, 20133 Milano, Italy
[3]Dipartimento di Fisica, Università di Pisa, 56127 Pisa, Italy
[4]Departamento de Física, Universidade Federal de Pernambuco, 50670-901 Recife-PE, Brazil
[5]IFN-CNR, Istituto di Fotonica e Nanotecnologie, 20133 Milano, Italy
[6]School of Physics and Astronomy, Monash University, Melbourne, VIC, 3800, Australia
[7]Department of Physics, Imperial College London, London, SW7 2AZ, United Kingdom
[8]Institute of Photonics, Hamburg University of Technology, 21073 Hamburg, Germany

Email: * A.Antonov@physik.uni-muenchen.de / † Luca.Sortino@physik.uni-muenchen.de / ‡ Andreas.Tittl@physik.uni-muenchen.de

Contents

1 Moving from Silicon to hBN 2

2 List of structural Parameters 3

3 Trapezoidal Unit Cell with Relative Asymmetry 4

4 Impact of Different Geometric Parameters 5

5 Introduction of Scaling Factor 7

6 Numerical Investigation of Strong Coupling 8

7 Reciprocity-Based Simulation of Photoluminescence Spectra 8

8 Optical Manipulation 10

9 Illustration of Fabrication Workflow 14

10 Sketch of Experimental Setup 15

1 Moving from Silicon to hBN

The radial qBIC structure based on silicon features a radius of approximately 1.5 µm and exhibits a resonance slightly above 700 nm. [1] In a first step, we gradually decrease the refractive index of the resonators, which leads to a blue-shift of the resonance and a reduction in modulation depth (Fig. S1a). Both effects arise from the reduced refractive-index contrast between the resonator and the substrate and are mitigated by uniformly upscaling the entire ring geometry. Increasing the ring radius red-shifts the resonance, while enlarging the resonator dimensions and the number of unit cells increases the total resonator volume, partially compensating for the lower index contrast. A design scaled by a factor of approximately 1.8 compared to the original silicon structure yields sufficiently strong resonances near 700 nm at a refractive index of $n = 2.6$, comparable to that of TiO_2. Further upscaling by a factor of 1.5, along with additional optimization steps (such as unit cell geometry, see Fig. 1), results in qBIC resonances with improved modulation depth and higher Q-factors than the initial silicon-based design.

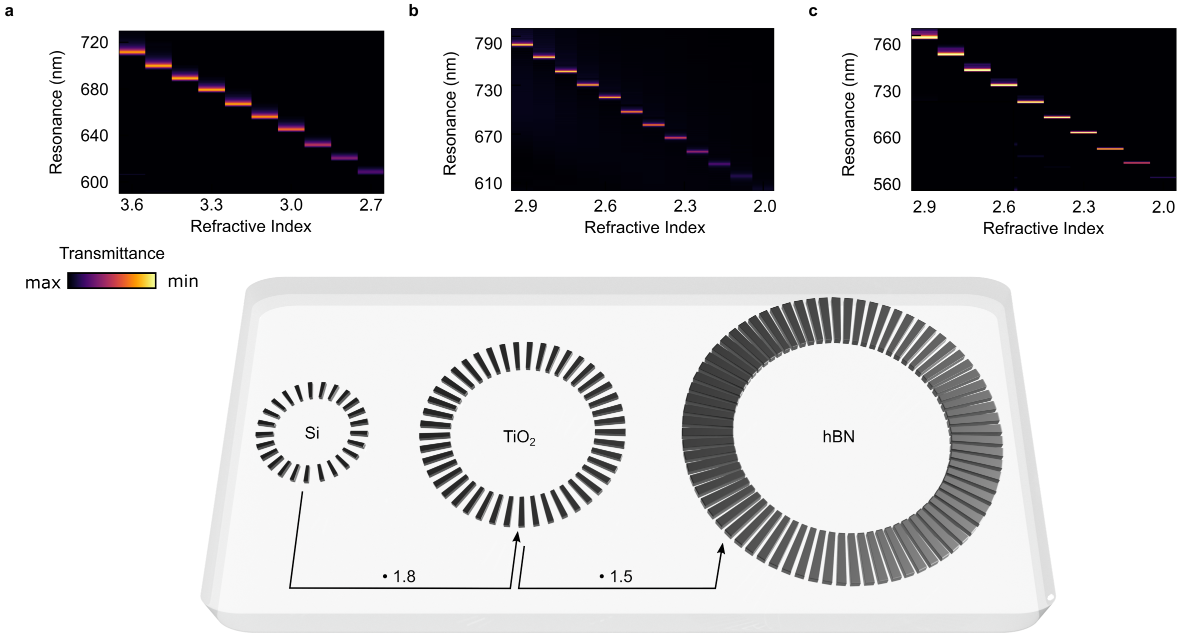

Figure S1: Moving from high refractive index dielectric to low refractive-index. Simulated transmittance for differently scaled radial qBIC structures, starting from the silicon design proposed in literature. [1] **a** The transition from a resonator material with a high refractive index ($n = 3.45$, silicon) to a low-index material such as hBN ($n_{x,y} = 2.1, n_z = 1.6$ in the investigated spectral range) also entails a reduction in refractive-index contrast between the resonators and the substrate (fused silica, $n = 1.45$). **b** Therefore, to attain sufficiently strong signal, the dimensions of the ring must first be increased by a factor of approximately 1.8 to recover a qBIC signal around 700 nm for a refractive index similar to that of TiO_2 ($n = 2.6$). **c** To achieve a comparable signal for hBN, the dimensions of the structure must be increased again by a factor of 1.5.

2 List of structural Parameters

Table S1: Geometrical parameters of the investigated radial qBIC metasurface configurations. For the experimentally fabricated samples, the listed values correspond to the nominal design parameters and not to post-fabrication measured dimensions.

Location	Type	R (nm)	Length (nm)	Gap (nm)	N	Height (nm)	Asymmetry
Fig. 1c (rods)	Simulation	3050	600	No constant gap; constant resonator width 150 nm	48	140	Varying absolute length asymmetry
Fig. 1c (trapezoids)	Simulation	3050	600	No constant gap; $w_{\text{inner}} = 120\,\text{nm}$ and $w_{\text{outer}} = 170\,\text{nm}$	48	140	Varying absolute length asymmetry
Fig. 1d	Simulation	3950	920	60 nm	68	165	$\Delta W = \Delta L = 20\%$
Fig. 1e	Simulation	3950	920	60 nm	68	165	$\Delta W = \Delta L = 20\%$
Fig. 1f (Width)	Experiment	3800	850	60 nm	68	150	$\Delta W = 30\%$
Fig. 1f (Double)	Experiment	3800	850	60 nm	68	150	$\Delta W = \Delta L = 30\%$
Fig. 2c	Simulation	3900	920	70 nm	68	165	$\Delta W = \Delta L = 20\%$
Fig. 2d	Simulation	3900	920	70 nm	68	165	$\Delta W = \Delta L = 20\%$
Fig. 2g	Simulation	3900	920	70 nm	68	165	$\Delta W = \Delta L = 20\%$
Fig. 2h	Simulation	3900	920	70 nm	68	165	$\Delta W = \Delta L = 20\%$
Fig. 3f	Experiment	1.0075·3900	1.0075·900	60 nm	68	160	$\Delta W = \Delta L = 20\%$
Fig. 3g	Experiment	1.0075·3900	1.0075·900	60 nm	68	140	$\Delta W = \Delta L = 20\%$
Fig. 4a	Experiment	1.02·3900	1.02·900	60 nm	68	140	$\Delta W = \Delta L = 20\%$
Fig. 4b	Experiment	1.02·3900	1.02·900	60 nm	68	140	$\Delta W = \Delta L = 20\%$
Fig. 4c	Experiment	1.02·3900	1.02·900	60 nm	68	140	$\Delta W = \Delta L = 20\%$
Fig. 4d	Experiment	1.01·3900	1.01·900	60 nm	68	140	$\Delta W = \Delta L = 20\%$
Fig. 4e	Experiment	1.01·3900	1.01·900	60 nm	68	140	$\Delta W = \Delta L = 20\%$

3 Trapezoidal Unit Cell with Relative Asymmetry

The exact calculation for the parallel side lengths of the trapezoids (W_{inner} and W_{outer}, w_1 and w_2 in the main text) in a symmetric radial qBIC ring uses the following parameters:

- N: number of unit cells (i.e., a pair of resonators). Each unit cell consists of two trapezoidal resonators, giving a total of $2N$ resonators in the ring.
- R: ring radius, defined as the distance from the ring center to the center of each trapezoid.
- l: trapezoid length, i.e., the distance between the two parallel sides.
- d: desired gap between neighboring resonators.

To simplify the calculation, the radial qBIC structure is approximated as a regular N-gon (polygon with N sides), instead of a continuous circular arc. The side lengths of the trapezoids are then given by:

$$W_{\text{inner}} = \frac{2\pi\left(R - \frac{l}{2}\right) - 2Nd}{2N}, \tag{S1}$$

$$W_{\text{outer}} = \frac{2\pi\left(R + \frac{l}{2}\right) - 2Nd}{2N}. \tag{S2}$$

Here, the term in parentheses represents the inner or outer circumference of the polygon, from which the total gap length is subtracted, and then normalized by dividing by the number of resonators.

Relative Asymmetry
To allow comparison between different radial qBIC designs, a relative asymmetry approach is introduced. Unlike absolute asymmetries, the relative asymmetry expresses differences in trapezoid dimensions as a fraction of the original symmetric dimensions, making designs with different overall scales directly comparable.

Let:

- dL_{rel}: relative length asymmetry
- dW_{rel}: relative width asymmetry

The absolute asymmetries are calculated from the symmetric design as:

$$dL = dL_{\text{rel}} \cdot l, \tag{S3}$$

$$dW_{\text{inner}} = dW_{\text{rel}} \cdot W_{\text{inner}}, \tag{S4}$$

$$dW_{\text{outer}} = dW_{\text{rel}} \cdot W_{\text{outer}}. \tag{S5}$$

To implement the asymmetries while maintaining constant gaps, the trapezoid widths are modified. First, the inner and outer widths of the longer resonators (those unaffected by the length asymmetry) are:

$$W_{\text{inner,long}} = \frac{2\pi\left(R - \frac{l}{2}\right) - 2Nd}{2N} + \frac{dW_{\text{inner}}}{2}, \tag{S6}$$

$$W_{\text{outer,long}} = \frac{2\pi\left(R + \frac{l}{2}\right) - 2Nd}{2N} + \frac{dW_{\text{outer}}}{2}. \tag{S7}$$

Then, for the shorter resonators (those affected by the length asymmetry), the corresponding widths are:

$$W_{\text{inner,short}} = \frac{2\pi\left(R - \frac{l}{2}\right) - 2Nd}{2N} - \frac{dW_{\text{inner}}}{2}, \tag{S8}$$

$$W_{\text{outer,short}} = \frac{2\pi\left(R + \frac{l}{2}\right) - 2Nd}{2N} - \frac{dW_{\text{outer}}}{2}. \tag{S9}$$

4 Impact of Different Geometric Parameters

Radius

This parameter allows for spectral tuning of the resonance (Fig. S2a) and can be conceptually compared to the length of a 1D chain. Increasing the radius leads to a red-shift of the resonance, while decreasing it results in a blue-shift. This trend is further influenced by the fact that, in the trapezoidal ring design, an increase in radius also increases the total resonator volume, since the resonator widths are continuously recalculated to maintain a constant total gap volume.

A key advantage of this design, compared to a rod-based approach, is that varying the radius over a reasonable range does not significantly impact the Q-factor of the resonance, thus broadening the design flexibility. This robustness is due to the constant gap width preserved by the trapezoidal geometry at any radius, in contrast to rod geometries where the gap width and especially the gap widening are radius-dependent. An explicit discussion of the gap parameter follows below.

Changes in modulation strength with varying radius can be attributed to the changing interaction cross-section between the incident light and the resonator ring.

Resonator Height

This parameter is predetermined by the thickness of the selected hBN flake. The modulation depth is strongly influenced by the resonator height, due to an increased volume available for optical field confinement within a high-index contrast environment. This increased confinement helps mitigate losses from substrate leakage, which explains the substantially reduced signal and Q-factor for thinner resonators (Fig. S2b).

Resonator Length

The resonator length refers to the center length of each trapezoid in the symmetric configuration. Increasing this length leads to a red-shift in the resonance position (Fig. S2c), primarily due to the corresponding increase in resonator volume. Additionally, a longer resonator increases the light–matter interaction cross-section, which manifests as stronger modulation in the transmittance spectrum.

Gap Width

The gap width is the fundamental parameter from which the resonator widths are calculated. A smaller gap results in stronger modulation and higher Q-factors, as it pushes the structure further into the sub-wavelength regime (Fig. S2d). The associated red-shift observed for narrower gaps is once again a result of increased resonator volume.

Since the resonator width is defined based on the gap width, the limit case of zero-width gaps leads to vanishing resonator width—at which point no mode confinement is possible, and the qBIC far-field response collapses.

Number of Unit Cells

Increasing the number of unit cells increases the total number of resonators and, consequently, the number of gaps. Because the gap width is kept constant, increasing the number of resonators changes the ratio between resonator volume and gap volume (i.e., air), leading to a blue-shift in the resonance position. The modulation depth and Q-factor increase asymptotically with more unit cells, due to the higher density of potential light confinement sites (Fig. S2e).

However, this improvement eventually saturates. Beyond a certain point, further increasing the number of resonators causes their individual widths to approach zero, again preventing mode confinement and suppressing qBIC formation.

The main practical limitation on the number of resonators is mechanical stability. In fabrication, it has proven advantageous to avoid structures with dimensions below 70 nm to prevent collapse. This constraint places an upper bound on the number of unit cells in hBN rings resonant around 620 nm at approximately 68.

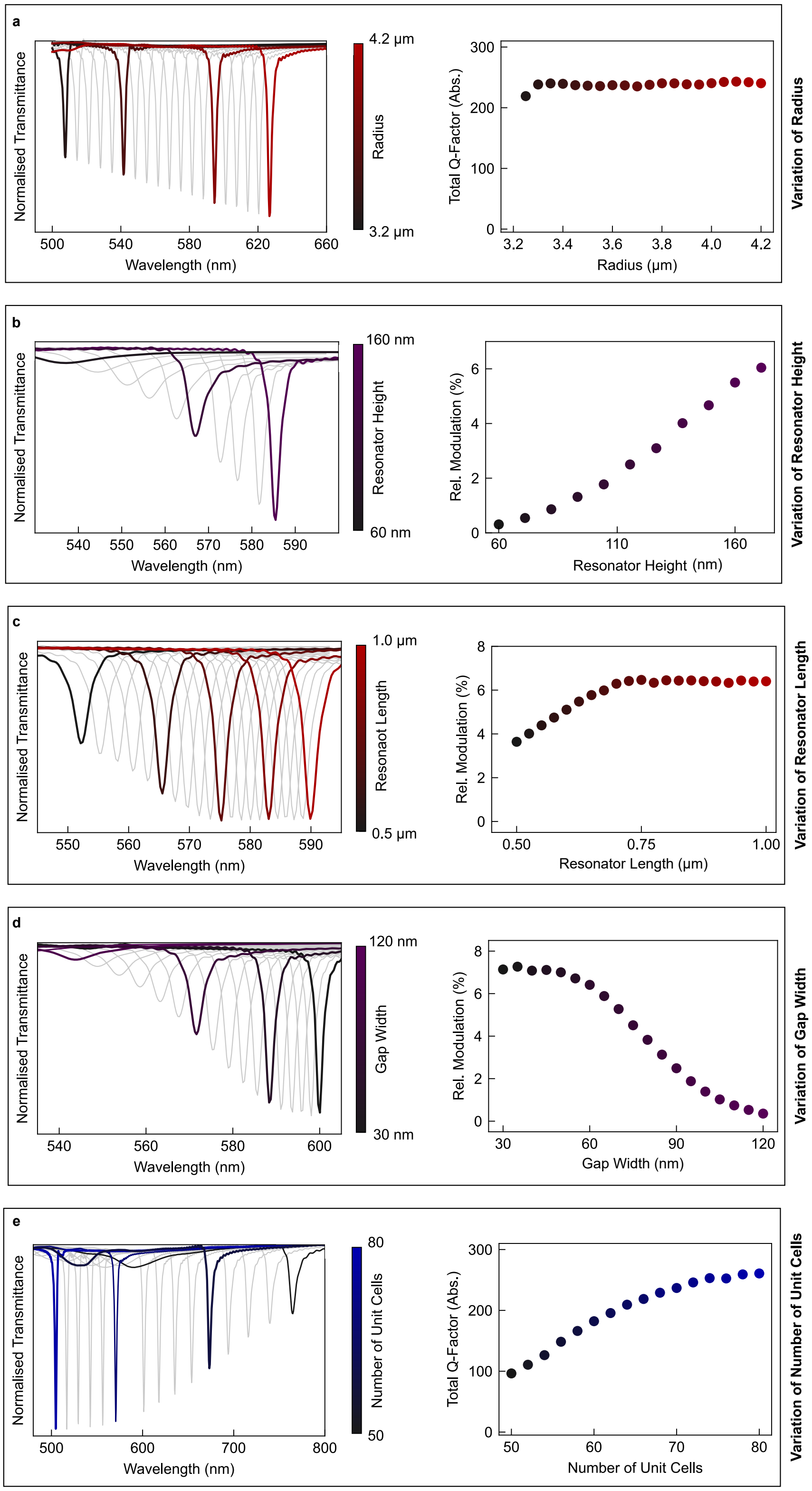

Figure S2: Impact of geometric parameters. Normalized simulated transmittance spectra for perturbation of individual geometric parameters while maintaining all others. The changing parameters are **a** Radius R, **b** Resonator Height h, **c** Resonator Length l, **d** Gap width d, **e** Number of unit cells N.

5 Introduction of Scaling Factor

In order to spectrally shift a radial qBIC over a small range, very minor steps in radius would be necessary (∼10 nm). Alternatively, the resonator length or the gap spacing can be changed on the scale of single nanometers. However, if the goal is to recreate a base radial qBIC's spectral response at a different spectral position, tuning only a singular parameter is a not fully optimized approach. Instead, the preferred approach in this work is to multiply every respective parameter of a radial qBIC by a scaling factor. Hence, all the fabricationally determinable parameters (radius, length, gap, and asymmetry) are multiplied by a scaling factor before running through the outlined calculation of the trapezoid widths. The only excluded parameters are the resonator height, which is assumed to be constant and predetermined by the exfoliated flake's height, and the unit cell number. This is due to the necessity of this always being an even and whole number; therefore, it is kept constant.

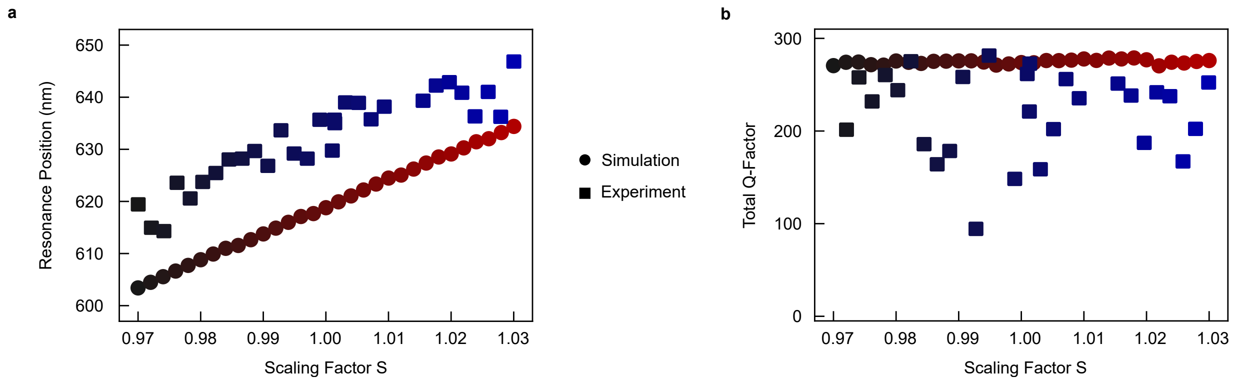

Figure S3: Introduction of Scaling Factor. Comparison of **a** resonance position and **b** total Q-factor for simulated and experimental spectra for a radial qBIC with a radius of 3.9 µm scaled with scaling factors ranging from 0.97 to 1.03.

The experimental and simulated implementation of this can be seen in Fig. S3, where in both cases a base ring of $R = 3.95$ µm radius, $l = 920$ nm resonator length, and $d = 60$ nm gaps is scaled linearly with scaling factors S ranging from 0.97 to 1.03 in a total of 30 equidistant steps. The unscaled parameters of unit cell and resonator height are $N = 68$ and $h = 165$ nm, respectively. The simulated results match the desired continuous and linear shift of resonance position with constant Q-factor. The experimental data follows the same trend in resonance position but with a red-shifted baseline, most likely due to anisotropic etching yielding an unaccounted-for increase in resonator volume. The quality factors of the experimental radial qBICs vary between 100 and 300. Nevertheless, the implementation of such a scaling factor allows very accurate shifting of the resonance of separate radial qBICs across a desired spectral range while maintaining the intended resonance lineshape. Furthermore, a substantial number of independently nanoscale-tuned parameters are consolidated into a singular tuning parameter, allowing for more straightforward analysis and discussion.

6 Numerical Investigation of Strong Coupling

Spectral coincidence such as in main text Fig. 3f points to the possibility of exciton-photon hybridization in the radial qBIC heterostructure system. A simplified energy level diagram (Fig. S4a) illustrates the conceptual formation of hybrid polariton states arising from coherent mixing between the exciton and qBIC mode. To explore this effect, we perform numerical simulations of the transmittance derivative (dT/dE) as a function of structural scaling (Fig. S4b). The resulting spectral dispersion exhibits an anticrossing near the exciton energy, a feature consistent with the formation of hybrid light-matter states. These results demonstrate the potential of radial qBIC resonantors to generate self-hybridized exciton-polaritons, opening the door for future experimental studies of strong coupling in such radially symmetric systems.

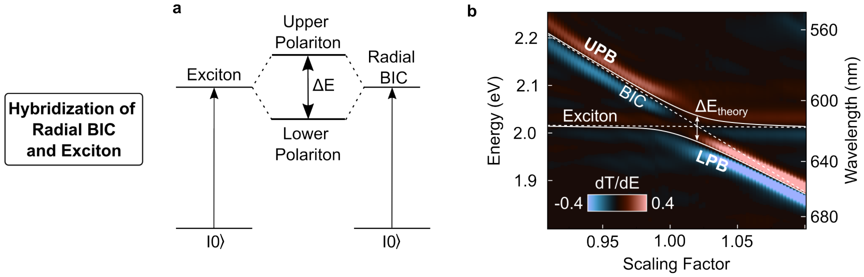

Figure S4: Strong Coupling. **a** Simplified energy level diagram of self-hybridization of excitons and radial BIC into polaritons. **b** Derivative of the transmittance (dT/dE) for simulated scaling increase showing anticrossing behavior.

7 Reciprocity-Based Simulation of Photoluminescence Spectra

To estimate the photoluminescence (PL), we employ the electromagnetic reciprocity principle, which allows the source and detector of electromagnetic fields to be interchanged without altering the physical situation. Consequently, the far-field radiation power and polarization originating from an ensemble of dipolar emitters can be evaluated by calculating the local field enhancements at the dipole positions under external illumination [2, 3]. The reciprocity principle ensures that evaluating the field enhancement within the emission structure yields qualitatively the same result as directly simulating dipole emission. The reciprocity relation used in our simulations follows from the Lorentz reciprocity theorem,

$$\int_V \mathbf{j}_1 \cdot \mathbf{E}_2 \, dV = \int_V \mathbf{j}_2 \cdot \mathbf{E}_1 \, dV, \tag{S10}$$

where $\mathbf{E}_{1,2}$ denote the electric fields generated by current densities $\mathbf{j}_{1,2}$, respectively.
For point dipole sources $\mathbf{p}_m$ located at positions $\mathbf{r}_m$ with

$$\mathbf{j}_m = -i\omega \mathbf{p}_m \, \delta(\mathbf{r} - \mathbf{r}_m), \quad m = 1, 2, \tag{S11}$$

the reciprocity relation reduces to

$$\mathbf{p}_1 \cdot \mathbf{E}_2(\mathbf{r}_1) = \mathbf{p}_2 \cdot \mathbf{E}_1(\mathbf{r}_2). \tag{S12}$$

Accordingly, in Fig. S5 we present the simulated intensity enhancement factor spectra and correlate it with the PL using this reciprocity-based approach.

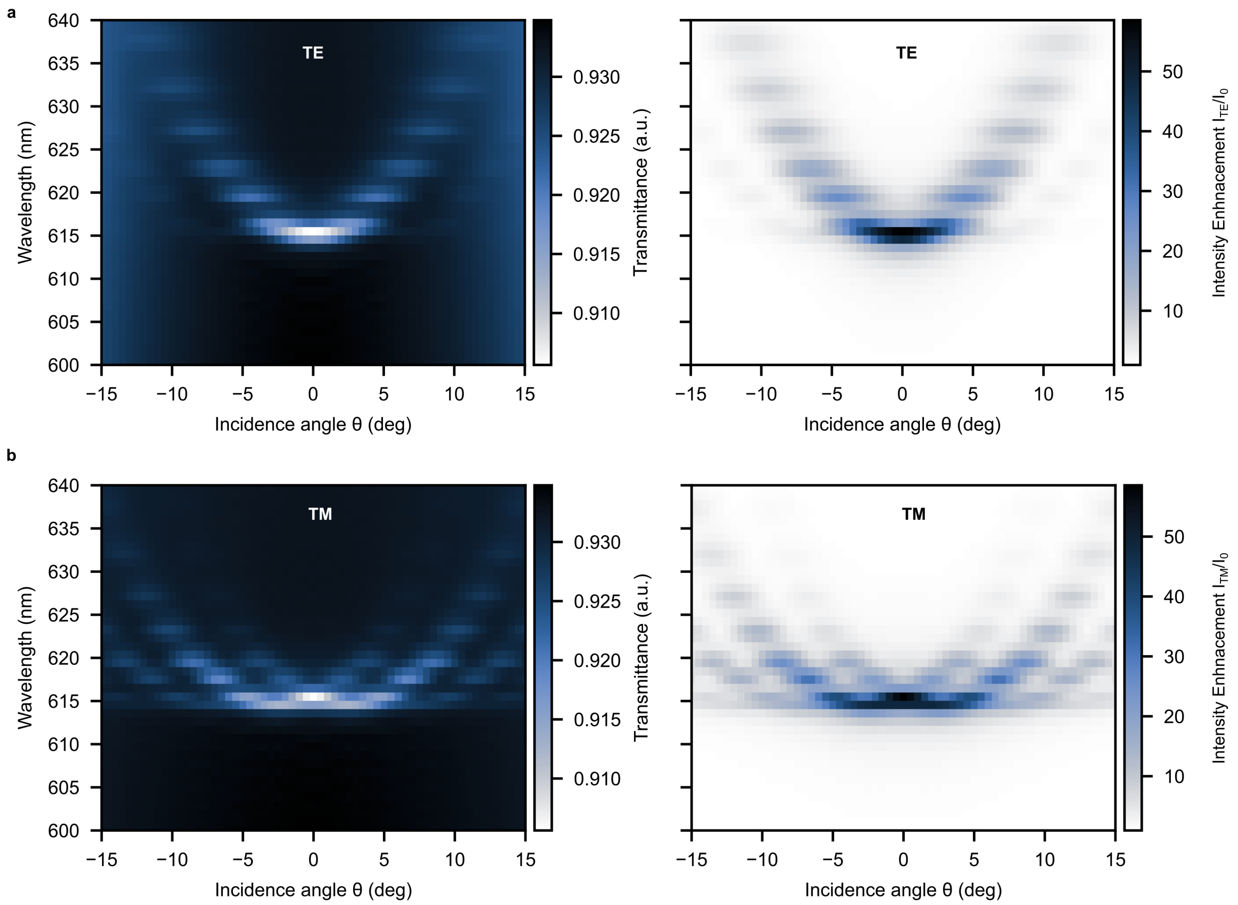

Figure S5: Comparison of simulated transmittance (left panel) and intensity enhancement factor within the ring metasurface (right panel). Angle- and wavelength-resolved maps are shown for **a** TE and **b** TM polarization.

8 Optical Manipulation

We propose an all-optical route to control the momentum-space response of our hBN-based radial qBIC platform by transiently modifying the local refractive index with an ultrafast pump. This concept is derived from the recent demonstration of temporally symmetry-broken metasurfaces by Aigner *et al.* [4], who achieve ultrafast radiative-loss control (resonance creation/annihilation, linewidth tuning) by selectively pumping a Mie resonance confined only in a part of an unit cell, which locally reduces the metasurface refractive index. Translating such a pumping-induced refractive index modulation mechanism to hBN (Fig. S6a) requires additional considerations, given that pristine hBN is a wide-gap insulator with an indirect bandgap close to $\sim$6 eV, placing its fundamental interband transitions in the deep ultraviolet (UV) [5]. Efficient interband excitation near the gap therefore typically requires deep-UV pump photons and specialized optical components (e.g., optics and sources) which present additional challenges. As a result, one would not generally expect strong free-carrier accumulation or large carrier-induced index modulation (e.g., $\Delta n \sim -0.13$ at 100 μJ cm^{-2} fluence pump as in silicon [4]) using visible/NIR pumps alone. Nevertheless, experimentally measurable pump-induced refractive-index nonlinearities in hBN have been observed under femtosecond excitation in the visible, manifesting as nonlinear refraction and nonlinear absorption [6], which indicate that sub-bandgap optical fields could nevertheless perturb the refractive index.

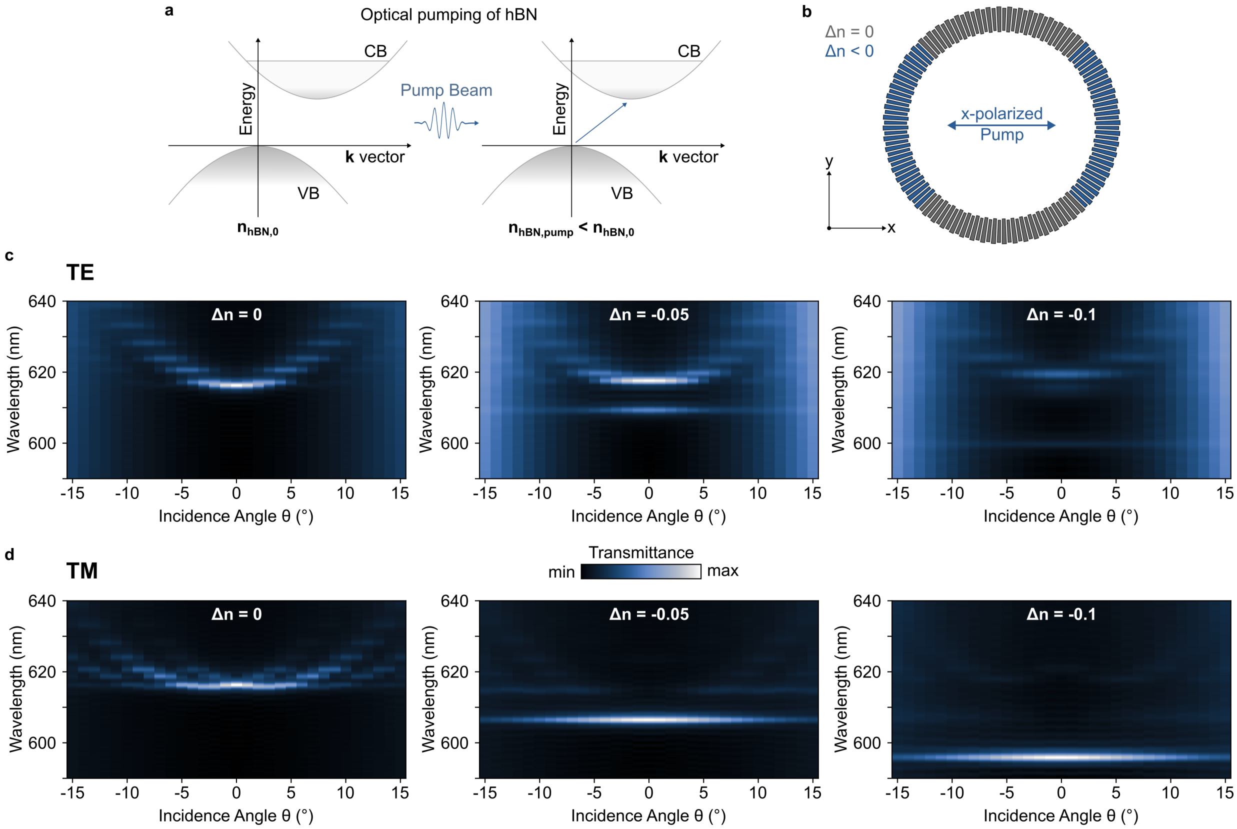

Figure S6: Pump-Polarization controlled refractive index modulation and momentum-space reconfiguration. We assume an unperturbed, isotropic refractive index of $n = 2.1$. The structural parameters of the radial BIC metasurface are identical to those employed in Figs. 2c and 2d of the main text. **a** Schematic illustration of optical pumping of hBN. A pump beam induces reduction of the refractive index ($\Delta n < 0$), resulting in $n_{\mathrm{hBN,pump}} < n_{\mathrm{hBN,0}}$. **b** Under x-polarized pumping, the induced refractive index change is assumed to be predominantly localized in rods oriented largely parallel to the pump polarization. **c** TE-polarized (light inclined in xz-plane) and **d** TM-polarized (light inclined in yz-plane) angle-resolved transmittance maps for increasing induced refractive index change ($\Delta n = 0,\ -0.05,\ -0.1$).

As illustrated in Fig. S6a, we consider optical pumping as a mechanism to transiently perturb the dielectric response of the metasurface. In the sub-bandgap regime, intense femtosecond excitation can modify the complex refractive index of hBN through nonlinear refraction and multiphoton-assisted excitation pathways [6], resulting in a refractive index change Δn. Pumping in the visible or longwave-UV, i.e., spectrally close to the metasurface's resonance, is expected to enable spatially selective modulation due to the polarization-selective nature of the elongated nanorod resonators. For simplicity, Δn is taken to be spatially uniform within the quadrants centered along the pump polarization and zero in the orthogonal quadrants. This idealized anisotropic perturbation captures the expected polarization-selective excitation while avoiding unnecessary spatial complexity. To connect the pump-induced momentum-space reconfiguration to the underlying ring eigenmodes, we additionally compute the complex eigenfrequencies and eigenfields of the unpumped and pumped structure (Fig. S7). For these eigenmode calculations, the substrate was omitted in order to reduce the computational cost of the FEM simulations. As a consequence, the absolute resonance wavelengths in Fig. S7a-c are slightly shifted with respect to those shown in Fig. S6c-d, where the full substrate-supported geometry was considered.

For TE polarization (Fig. S6c), the unpumped case ($\Delta n = 0$) exhibits the characteristic WGM-like dispersion already shown in the main text, i.e., a curved resonance branch centered around Γ that follows the expected angular dependence of a circulating photonic mode. The ring-like standing-wave modulation in the eigenfields further supports the assignment of these dispersive features to circulating WGM-like ring modes (Fig. S7d). Upon introducing a moderate negative index perturbation ($\Delta n = -0.05$), a second feature emerges: a flat spectral branch develops, coexisting with the residual WGM-like dispersion. This indicates that the anisotropic perturbation partially lifts the azimuthal symmetry and redistributes the radiative coupling across momentum space. For stronger modulation ($\Delta n = -0.1$), the flatband contribution becomes more pronounced and spectrally separated, while remnants of the curved WGM-like branch remain visible. Hence, in TE polarization, optical pumping enables a regime in which WGM-dominated and flatband-like momentum-space responses coexist and can be continuously tuned through the pump-induced Δn.

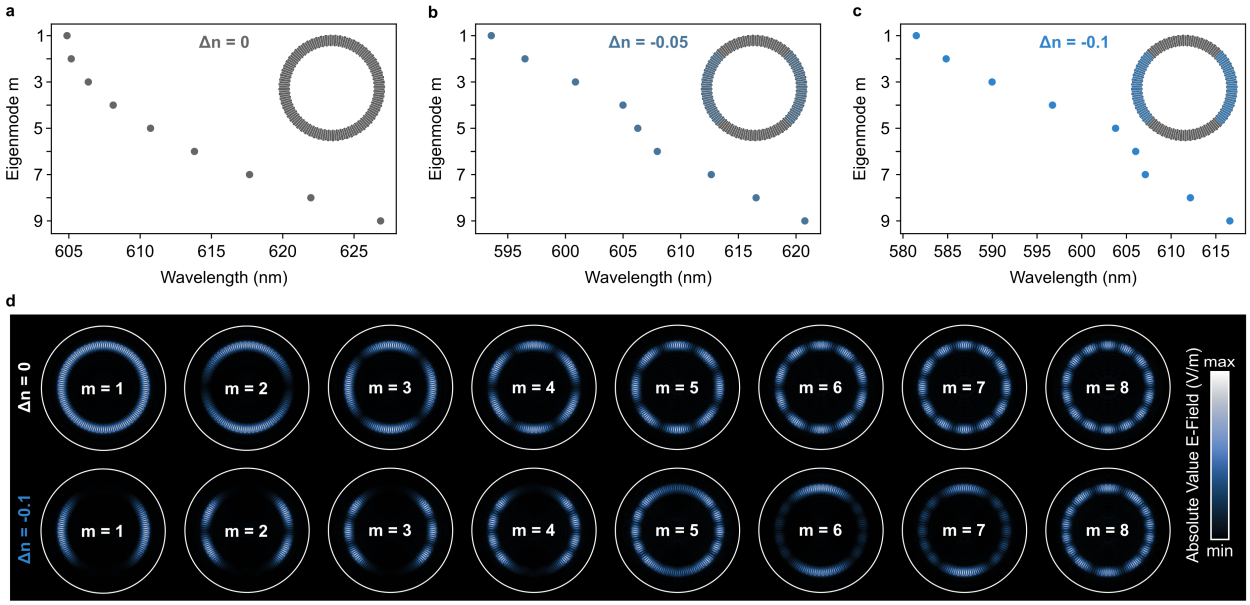

Figure S7: Eigenfields of the radial metasurface with pump-controlled refractive index modulation. We assume an unperturbed, isotropic refractive index of $n = 2.1$. The structural parameters of the radial BIC metasurface are identical to those employed in Figs. 2c and 2d of the main text (omitting substrate). Spectral position of the eigenmodes for **a** $\Delta n = 0$, **b** $\Delta n = -0.05$ and **c** $\Delta n = -0.1$. **d** Absolute value of the E-field for the first eight eigenmodes (mode number 1 to 8) of the radial qBIC structure for $\Delta n = 0$ and $\Delta n = -0.1$.

In contrast, the TM response (Fig. S6d) shows a more abrupt reconfiguration. In the unperturbed structure,

the dispersion is likewise dominated by a curved WGM-like branch. However, already for $\Delta n = -0.05$, this angular dispersion collapses into a nearly dispersionless resonance around Γ, forming a flatband that spans the radiatively accessible angular range. Increasing the modulation to $\Delta n = -0.1$ further stabilizes this flat response, while the original WGM-like curvature is effectively suppressed. In this polarization channel, the pump-induced symmetry breaking therefore enables an all-optical ultrafast switching between a momentum-space response governed by circulating WGM-like character and a flatband-dominated regime. At the level of this simplified model, the emergence of a flat branch is consistent with the fact that the pump breaks the effective rotational uniformity of the ring by modifying only a subset of resonators. Such an anisotropic perturbation is expected to alter the phase-matching conditions for coupling between free-space plane waves and the discrete ring eigenmodes mediated by the qBIC. As a result, the angular dependence of the radiative coupling can be strongly reduced, yielding a comparatively flat spectral response versus incidence angle. The stronger reconfiguration observed for TM compared to TE indicates that the TM channel is more sensitive to this quadrant-selective perturbation, in line with the general polarization dependence of oblique-incidence excitation discussed in the main text (Fig. 2). We emphasize that these simulations are intended as a proof-of-concept for momentum-space reshaping via ultrafast index modulation; identifying the exact origin of the flat response would require additional analysis beyond the scope of this section.

A closer inspection of the eigenfield evolution furthermore reveals a mode-selective response to the pump-induced index perturbation. For $\Delta n = -0.1$, the lowest-order modes ($m = 1 - 4$) retain the characteristic WGM-like standing-wave modulation observed in the unperturbed structure, but their intensity becomes predominantly confined to the pumped quadrants (Fig. S7d). This indicates that the circulating character of these modes persists, while the spatial distribution adapts to the anisotropic index. In addition, the low-order modes become spectrally less dense. Beginning around $m \approx 6$, the modal character changes qualitatively. The corresponding eigenfield resembles a 90°-rotated version of the lowest-order pumped mode with vanishing WGM features, rather than a simple continuation of the WGM-like sequence. Notably, this transition coincides with a visible change in the spectral evolution of the eigenfrequencies: whereas the unperturbed structure exhibits a nearly monotonic progression with increasing mode index, the pumped cases (particularly $\Delta n = -0.1$) display a distinct deviation around this mode number (Fig. S7a-c). For higher-order modes ($m \gtrsim 8$), however, the eigenfield patterns of the pumped and unperturbed structures become increasingly similar again. The combination of rotational symmetry and qBIC mediated radiation channels in the radial qBIC platform uniquely enables pump-induced control over the hierarchy of WGM eigenmodes. Such dynamic restructuring of circulating photonic states opens opportunities for ultrafast manipulation of OAM-selective emission, momentum-space routing, and symmetry-controlled light-matter interactions.

Structured Light Excitation

To investigate selective excitation of the ring eigenmode ladder, we numerically excite the radial qBIC with vortex beams carrying orbital angular momentum (OAM) ℓ at normal incidence. The incident field is implemented as an imported Laguerre-Gaussian-like mode with radial index $p = 0$, azimuthal phase dependence $e^{i\ell\phi}$, and a Gaussian radial envelope. The beam waist is chosen such that the intensity maximum approximately overlaps with a target ring radius $R_{\text{target}} = 4.5\ \mu\text{m}$, corresponding to the outer radius of the patterned structure used in the simulations. For increasing $|\ell|$, the effective waist is scaled as $w_{\text{eff}} = R_{\text{target}}\sqrt{2/|\ell|}$ to maintain overlap between the annular intensity distribution and the resonator ring. The field is linearly polarized (x-polarized in the simulations) and normalized to constant peak amplitude for all ℓ. Broadband excitation is applied in the range $\lambda \in [620, 660]$ nm, and the Γ-point transmittance is extracted from a transmission monitor and normalized to the incident power. As the geometry and material parameters remain unchanged, the eigenfrequencies of the structure are independent of excitation; the observed ℓ-dependent spectral evolution therefore reflects selective coupling to different eigenmodes rather than a shift of a single resonance. Due to computational cost, the wavelength sampling is limited, and the spectra therefore do not resolve very narrow features. As shown in Fig. S8, OAM excitation reveals one or, for certain ℓ, two distinct resonant dips directly at Γ, with increasing spectral separation for larger $|\ell|$. This behavior is consistent with the picture developed in the main text (Fig.2), where the radial qBIC mediates radiative access to a discrete ladder of ring eigenmodes carrying azimuthal order m. The structured phase profile enables direct coupling to these angular modes at normal incidence, whereas under plane-

wave excitation they are primarily resolved in angle-resolved (k-space) measurements. Because the beam is normalized to constant peak amplitude while the waist decreases with increasing $|\ell|$, the effective spatial extent of the excitation becomes progressively smaller. In the present study we therefore restrict the analysis to $|\ell| \leq 4$. Here, the target radius $R_{\text{target}} = 4.5\ \mu$m denotes the radial position of the intensity maximum of the imported vortex beam and is chosen to approximately match the outer radius of the patterned ring (i.e., the beam is aligned to the outer extent of the resonator annulus). For $\lambda_0 = 620$ nm and $R_{\text{target}} = 4.5\ \mu$m, the effective waist for $|\ell| = 4$ is $w_{\text{eff}} \approx 3.2\ \mu$m ($\approx 5\,\lambda_0$). The corresponding annular intensity width remains several wavelengths wide, and the estimated beam divergence $\theta_{\text{div}} \sim \lambda_0/(\pi w_{\text{eff}}) \approx 3.5^\circ$ indicates that the excitation remains well within the paraxial regime. For substantially larger $|\ell|$, however, the decreasing waist would lead to a strongly confined annular profile with increased transverse wavevector content, such that the excitation would no longer represent a well-defined paraxial OAM mode.

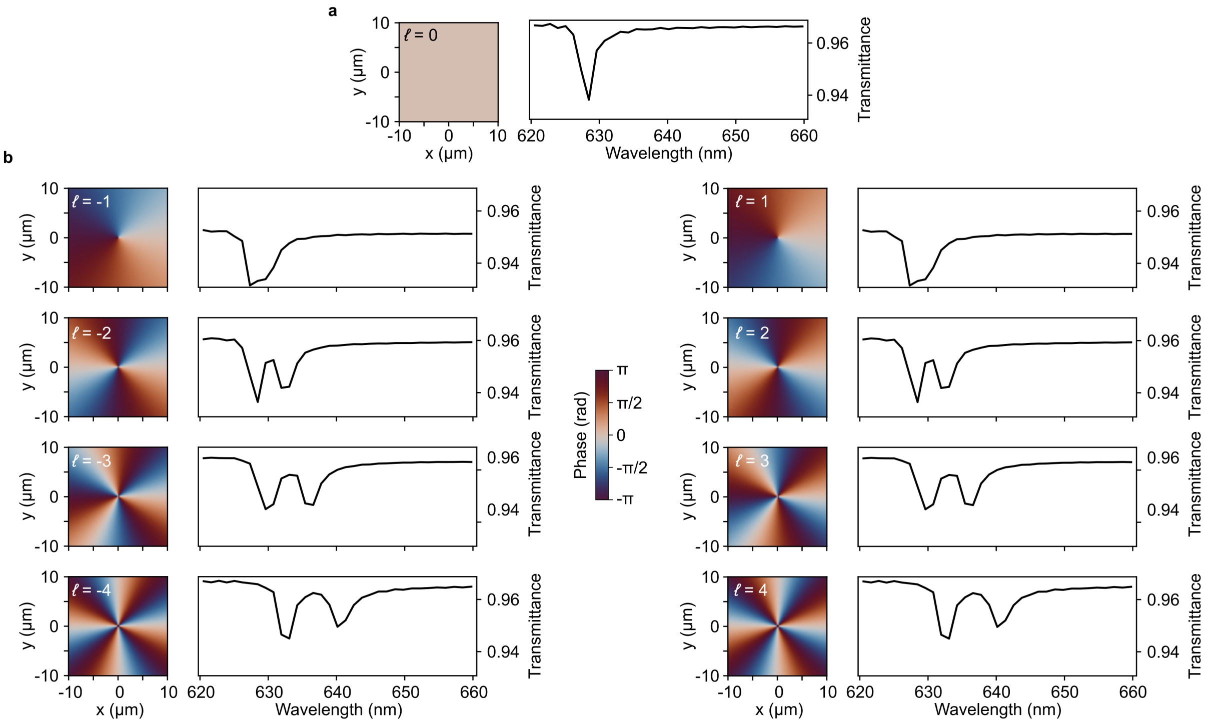

Figure S8: OAM-selective excitation at normal incidence. Calculated Γ-point transmittance of the radial qBIC metasurface under vortex-beam excitation with orbital angular momentum ℓ. The left panels show the wrapped phase of the incident field, the right panels the corresponding transmittance spectra. **a** While plane-wave excitation ($\ell = 0$) yields a single radiative resonance, **b** OAM excitation reveals one or two distinct dips at Γ-point, with increasing spectral separation for larger $|\ell|$. As the structure is unchanged, the ℓ-dependent response reflects selective coupling to different azimuthal ring eigenmodes mediated by the qBIC, which under plane-wave excitation are only resolved in k-space.

9 Illustration of Fabrication Workflow

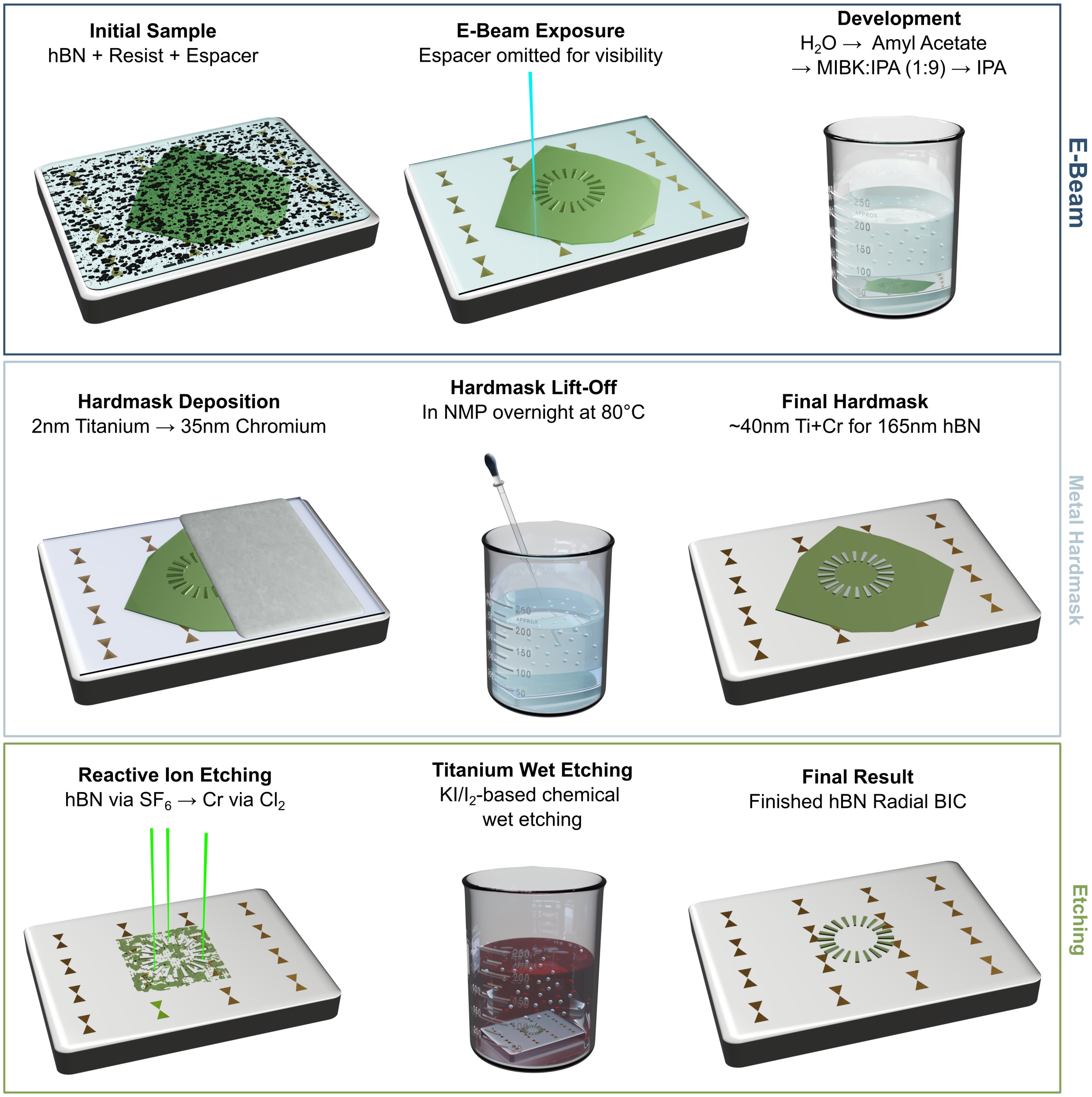

Figure S9: Fabrication Workflow. Graphic outline of the workflow for e-beam exposure, subsequent hardmask deposition and etching resulting in final hBN radial qBIC platform.

10 Sketch of Experimental Setup

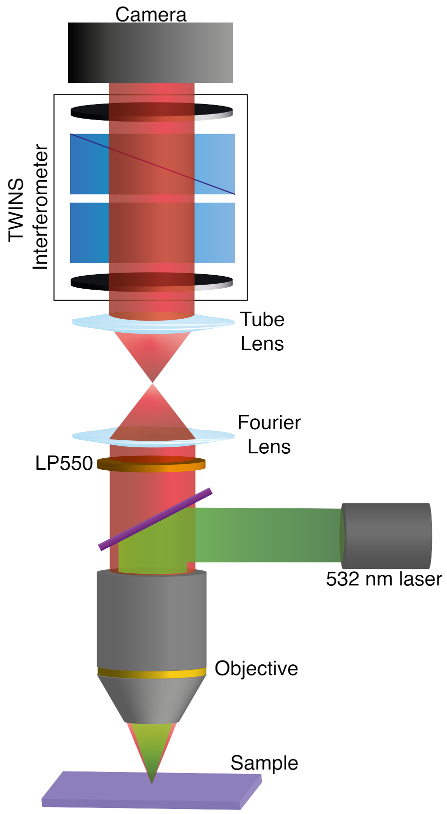

Figure S10: Sketch of the Fourier-space hyperspectral microscope. A 532 nm CW laser is used to illuminate the sample, filtered by a dichroic mirror and long-pass filter. A 100× objective (NA = 0.75) collects the emission. A Fourier lens and TWINS interferometer image the back focal plane onto the camera. Details on the technique can be found in [7, 8].

References

(1) Kühner, L.; Sortino, L.; Berté, R.; Wang, J.; Ren, H.; Maier, S. A.; Kivshar, Y.; Tittl, A. Radial bound states in the continuum for polarization-invariant nanophotonics. *Nature Communications* **2022**, *13*, 4992.

(2) Maksimov, A.; Tartakovskii, I.; Filatov, E.; Lobanov, S.; Gippius, N.; Tikhodeev, S.; Schneider, C.; Kamp, M.; Maier, S.; Höfling, S.; Kulakovskii, V. D. Circularly polarized light emission from chiral spatially-structured planar semiconductor microcavities. *Physical Review B* **2014**, *89*, 045316.

(3) Kim, S.; An, S.-C.; Kim, Y.; Shin, Y. S.; Antonov, A. A.; Seo, I. C.; Woo, B. H.; Lim, Y.; Gorkunov, M. V.; Kivshar, Y. S.; Kim, J. Y.; Jun, Y. C. Chiral electroluminescence from thin-film perovskite metacavities. *Science Advances* **2023**, *9*, eadh0414.

(4) Aigner, A.; Possmayer, T.; Weber, T.; Antonov, A. A.; de S. Menezes, L.; Maier, S. A.; Tittl, A. Optical control of resonances in temporally symmetry-broken metasurfaces. *Nature* **2025**, *644*, 896–902.

(5) Cassabois, G.; Valvin, P.; Gil, B. Hexagonal boron nitride is an indirect bandgap semiconductor. *Nature Photonics* **2016**, *10*, 262–266.

(6) Ouyang, Q.; Zhang, K.; Chen, W.; Zhou, F.; Ji, W. Nonlinear absorption and nonlinear refraction in a chemical vapor deposition-grown, ultrathin hexagonal boron nitride film. *Optics Letters* **2016**, *41*, 1368–1371.

(7) Brida, D.; Manzoni, C.; Cerullo, G. Phase-locked pulses for two-dimensional spectroscopy by a birefringent delay line. *Optics Letters* **2012**, *37*, 3027–3029.

(8) Genco, A.; Cruciano, C.; Corti, M.; McGhee, K. E.; Ardini, B.; Sortino, L.; Hüttenhofer, L.; Virgili, T.; Lidzey, D. G.; Maier, S. A.; Bassi, A.; Valentini, G.; Cerullo, G.; Manzoni, C. k-Space hyperspectral imaging by a birefringent common-path interferometer. *ACS Photonics* **2022**, *9*, 3563–3572.
